# Comparative outcomes of heart failure among existent classes of anti-diabetic agents: a network meta-analysis of 171,253 participants from 91 randomized controlled trials

**DOI:** 10.1186/s12933-019-0853-x

**Published:** 2019-04-08

**Authors:** Da-ya Yang, Xin He, Hui-wei Liang, Shao-zhao Zhang, Xiang-bin Zhong, Chu-fan Luo, Zhi-min Du, Jian-gui He, Xiao-dong Zhuang, Xin-xue Liao

**Affiliations:** 1 Department of Cardiology, First Affiliated Hospital of Sun Yat-Sen University, No. 58 Zhong Shan 2nd Road, Guangzhou, 510080 China; 2 NHC Key Laboratory of Assisted Circulation, Sun Yat-Sen University, Guangzhou, China; 3 Administrative Office of Clinical Trial Center, Guangzhou Hui-Ai Hospital, Guangzhou, China

**Keywords:** Cardiovascular, Meta-analysis, Heart failure, Diabetes, Agent

## Abstract

**Background:**

The cardiovascular (CV) safety in terms of heart failure among different classes of treatment remains largely unknown. We sought to assess the comparative effect of these agents on heart failure outcomes.

**Methods:**

This study was registered in the International Prospective Register of Systematic Reviews (CRD 42016042063). MEDLINE, EMBASE, and the Cochrane Library Central Register of Controlled Trials were searched. For the primary outcomes reported previously, studies between Jan 1, 1980 and June 30, 2016 were screened, and subsequently updated till Jan 24, 2019. We performed network meta-analysis to obtain estimates for the outcomes of heart failure, in particular by rankograms for ranking of heart failure risk as well as by pairwise comparisons among all classes of anti-diabetic medications.

**Results:**

A total of 91 trials were included, among which were 171,253 participants and 4163 reported cases of heart failure events. As for rankograms, the surface under the cumulative ranking curves (SUCRA) of sodium-glucose co-transporters 2 and thiazolidinediones were 93.4% and 4.3%, respectively, signifying the lowest and highest risk of heart failure, respectively. As for pairwise comparisons in the network, sodium-glucose co-transporters 2 were significantly superior to insulin (OR: 0.75, 95% CI 0.62–0.91), dipeptidyl peptidase 4 inhibitors (OR: 0.68, 95% CI 0.59–0.78), glucagon-like peptide-1 receptor agonists (OR: 0.65, 95% CI 0.54–0.78), and thiazolidinediones (OR: 0.46, 95% CI 0.27–0.77) in terms of heart failure risk. Furthermore, in an exploratory analysis among subjects with underlying heart failure or at risk of heart failure, the superiority of sodium-glucose co-transporters 2 was still significant.

**Conclusions:**

In terms of heart failure risk, sodium-glucose co-transporters 2 were the most favorable option among all classes of anti-diabetic medications.

**Electronic supplementary material:**

The online version of this article (10.1186/s12933-019-0853-x) contains supplementary material, which is available to authorized users.

## Introduction

In 1974, the Framingham studies have reported an approximately two- to five-fold increase for the risk of heart failure in patients with diabetes [1]. Rates of mortality and hospitalization from heart failure were also higher among diabetics than non-diabetics [2–5]. However, listed alongside with atherosclerotic cardiovascular (CV) diseases as one of the two major CV safety concerns [6], heart failure as a “frequent, forgotten, and often fatal complication of diabetes” [7] involves complex, multifactorial pathogenesis, and cannot be indiscreetly pigeonholed into the stereotypical, binary classification as either microvascular or macrovascular [8]. Further complicating the matter, glycemic control per se did not reduce the risk of heart failure [9], and might even adversely affect cardiac function in susceptible individuals [10–12]. The harmful effect of developing heart failure appeared to be associated with improved glucose control and was explained, at least in part, by choice of treatment, as indicated in a recent meta-analysis [13]. Whether or not anti-diabetic medications directly contribute to the progression and/or precipitation of heart failure, as well as which class of anti-diabetic medications is the optimal choice for this patient population, have henceforth been and are still being intensely debated and investigated [8, 14–21]. Previously, we have conducted a network meta-analysis to evaluate whether novel anti-diabetic agents, including dipeptidyl peptidase 4 (DDP-4) inhibitors, glucagon-like peptide-1 (GLP-1) receptor agonists, and sodium-glucose co-transporter 2 (SGLT-2) inhibitors, were superior in terms of major adverse cardiovascular events (MACE) and all-cause mortality, compared with more traditional classes of drugs [22]. Here, we performed a similar study aiming at providing evidence-based hierarchies of the comparative CV safety profiles, specifically regarding heart failure endpoints, among classes of anti-diabetic agents currently in use.

## Methods

This study was registered in the International Prospective Register of Systematic Reviews (CRD 42016042063). The systematic review and meta-analysis was conducted according to the Preferred Reporting Items of Systematic Reviews and Meta-Analyses (PRISMA) guideline [23]. The method and results of the primary outcomes were reported previously [22]. This study reported the secondary outcome of heart failure. The network meta-analysis was based on a frequentist model [24]. The analysis incorporated both direct and indirect comparisons to estimate the relative efficacy of treatments.

### Data source and study inclusion

MEDLINE, EMBASE, and the Cochrane Library Central Register of Controlled Trials were searched. For the primary outcomes reported previously, studies between Jan 1, 1980 and June 30, 2016 were screened. We updated the data until Jan 6, 2019 for the outcome of heart failure. Data on http://www.clinicaltrials.gov were also checked if the registry number was provided. The strategy of searching was provided in Additional file 1: Table S1.

To be included, studies had to: be randomized controlled trials; included individuals with type 2 diabetes; compared one kind of anti-diabetic with another or placebo; had at least 1 incident heart failure during follow-up; had a treatment duration of more than 24 weeks. Studies were excluded if they: were crossover studies; non-randomized trials; compared the different dosages or forms of the same drugs; enrolled patients with type 1 diabetes, prediabetes, or insulin resistance.

### Novel anti-diabetics and dosages

Novel anti-diabetics in our studies referred to the following 3 categories: dipeptidyl peptidase 4 inhibitors (DPP4i), glucagon-like peptide-1 receptor agonists (GLP1a), andsodium-glucose co-transporter 2 inhibitors (SGLT2i). We only included studies on drugs that were already approved by US FDA or European Medicines Agency. Data of the novel anti-diabetic treatment arm were excluded if the dosages were inconsistent with the recommended ones (Additional file 2: Table S2).

### Data extraction and quality assessment

Outcome of interest in this study was incident heart failure or hospitalization for heart failure. Data were extracted by 2 independent reviewers. Studies characteristics (registry number, name the first author, whether it was international study, number of study centers, and treatment duration), patients characteristics (mean age, male proportion, and comorbidity), intervention details (type and dosage of the studied drugs, and baseline anti-diabetic used across arms), total number of participants and incidence of heart failure.

The methodological quality of included studies was assessed using the tool recommended by Cochrane collaboration [25]. This tool included 7 components, which are random sequence generation, allocation concealment, blinding of participants and personnel, blinding of outcome assessment, incomplete outcome data, selective reporting and other sources of bias. Each of these components was rated as “high risk”, “unknown”, or “low risk”.

### Statistical analysis

Network meta-analysis was performed to compare any of the 2 anti-diabetics by incorporating direct and indirect comparison data. The results were reported as odd ratios (ORs) and 95% confidence intervals (CIs). We then generated a ranking of the anti-diabetics in terms of risk of heart failure by calculating the surface under the cumulative ranking curves (SUCRA). Larger SUCRA indicated lower risk of heart failure. Rankograms were also generated [26].

To identify any potential inconsistency between direct and indirect comparisons data, we used a previously described node-splitting method. It separates evidence for a particular comparison into direct and indirect component, excluded one direct comparison at a time, and estimated the indirect treatment effect for the excluded comparison [27].

All the analyses were performed using STATA version 13 with the *mvmeta* package.

## Results

Flow chart of study inclusion is shown in Additional file 3: Figure S1. Finally, 91 studies were included. There were totally 171,253 participants and 4163 reported cases of incident heart failure. Eight treatments were compared with each other: DPP4i, GLP1a, SGLT2i, Metformin (MET), sulfonylureas (SU), thiazolidinedione (TZD), insulin (INS), and placebo (PLA). Characteristics of studies and numbers of heart failure cases are summarized in Additional file 4: Table S3. Mean age of included studies ranged from 44.0 to 72.5 years. Proportion of male ranged from 41.3 to 77.6%. Three studies included patients with coronary heart disease. Nine studies included patients with high cardiovascular risk. Eight studies included patients with renal impairment. One study included patients with high cardiovascular and renal risk. Patients in these 23 studies were regarded as at high risk of heart failure and included in the exploratory analysis.

Assessment of methodological quality of included studies is summarized in Additional file 4: Table S4. The quality of included studies was generally good, but more than a half of the studies did not provide details about random sequence generation.

**Fig. 1:**
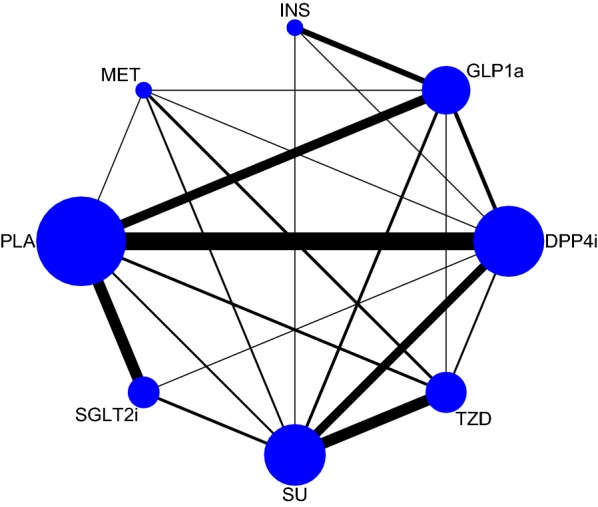
Network plot of treatment comparisons for heart failure. Direct comparison of treatment is linked with a line, the thickness of which represents the number of trials that assess the comparison. *SGLT2i* sodium-glucoseco-transporter 2 inhibitors, *GLP1ra* glucagon-like peptide-1 receptor agonists, *DPP4i* dipeptidyl peptidase 4 inhibitors, *TZD* thiazolidinediones, *MET* metformin, *SU* sulfonylureas, *INS* insulin, *PLA* placebo

Network plot of the comparisons is presented in Fig. 1. Direct comparisons were available for any of the 2 study arms, except for INS and MET, INS and PLA, INS and SGLT2i, INS and TZD, and SGLT2i and GLP1a.

**Fig. 2:**
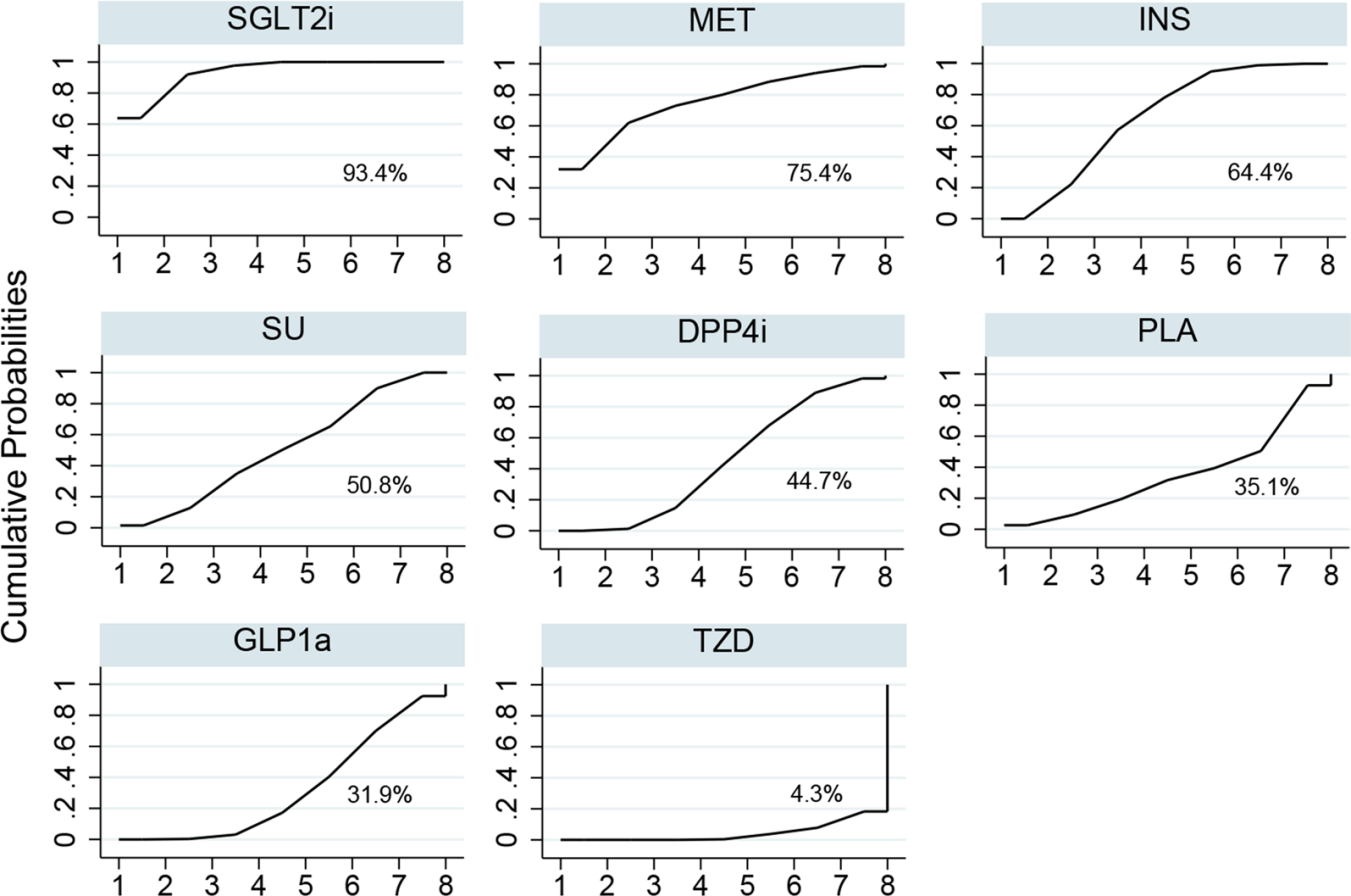
Ranking of anti-diabetic agents ordered by their respective probability to be the best treatment for heart failure endpoints. *SGLT2i* sodium-glucoseco-transporter 2 inhibitors, *GLP1ra* glucagon-like peptide-1 receptor agonists, *DPP4i* dipeptidyl peptidase 4 inhibitors, *TZD* thiazolidinediones, *MET* metformin, *SU* sulfonylureas, *INS* insulin, *PLA* placebo

Sample sizes and numbers of incident heart failure of included studies are summarized in Additional file 4: Table S5. Network plot of the comparisons is presented in Fig. 1. Direct comparisons were available for any of the 2 study arms, except for INS and MET, INS and PLA, INS and SGLT2i, INS and TZD, and SGLT2i and GLP1a. Rankograms and SUCRAs of the 8 treatments are summarized in Fig. 2. With the highest SUCRA of 93.4%, SGLT2i was associated with the lowest risk of heart failure compared with other drugs and PLA. In the contrast, TZD had the lowest SUCRA of 4.3%, which represented the highest risk of heart failure. MET, INS, SU, DPP4i, PLA, and GLP1a ranked 2nd to 7th, respectively.

**Fig. 3:**
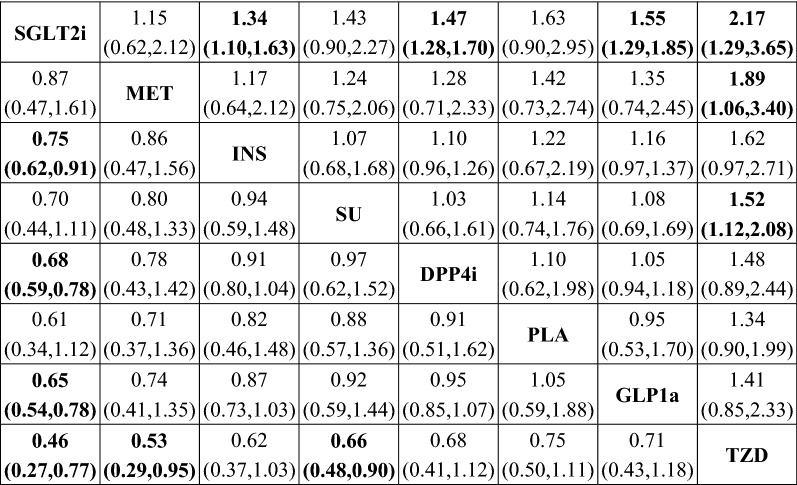
Mixed comparisons of anti-diabetic agents for heart failure by drug class. Agents are reported in order of heart failure risk ranking. Treatment at the top left corner ranks first, while the one at the bottom right corner ranks last. An odds ratio (OR) lower than 1 indicates better safety for heart failurein favor of the column-defining treatment. *SGLT2i* sodium-glucoseco-transporter 2 inhibitors, *GLP1ra* glucagon-like peptide-1 receptor agonists, *DPP4i* dipeptidyl peptidase 4 inhibitors, *TZD* thiazolidinediones, *MET* metformin, *SU* sulfonylureas, *INS* insulin, *PLA* placebo

**Fig. 4:**
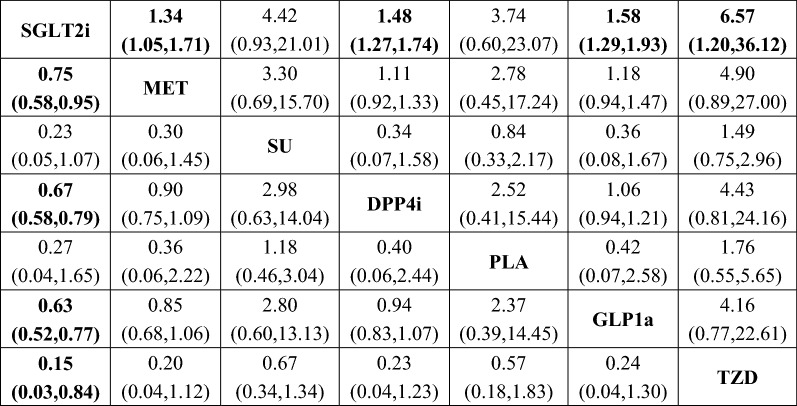
Mixed comparisons of anti-diabetic agents used among patients at high risk of heart failure by drug class. Agents are reported in order of heart failure risk ranking. Treatment at the top left corner ranks first, while the one at the bottom right corner ranks last. An odds ratio (OR) lower than 1 indicates better safety for heart failure in favor of the column-defining treatment. *SGLT2i* sodium-glucoseco-transporter 2 inhibitors, *GLP1ra* glucagon-like peptide-1 receptor agonists, *DPP4i* dipeptidyl peptidase 4 inhibitors, *TZD* thiazolidinediones, *MET* metformin, *SU* sulfonylureas, *PLA* placebo

Results of the network meta-analysis were summarized in Fig. 3. SGLT2i was significantly superior to INS (OR: 0.75, 95% CI 0.62–0.91), DPP4i (OR: 0.68, 95% CI 0.59–0.78), GLP1a (OR: 0.65, 95% CI 0.54–0.78), and TZD (OR: 0.46, 95% CI 0.27–0.77) in terms of heart failure risk. In addition, a lower risk than TZD was also found for MET (OR: 0.53, 95% CI 0.29–0.95) and SU (OR: 0.66, 95% CI 0.48–0.90). No significant inconsistency was identified (Additional file 5: Table S6). Results of the exploratory analysis were presented in Fig. 4. Superiority of SGLT2i, when compared with DPP4i, GLP1a, and TZD, was still significant. In this subpopulation, SGLT2i was even more effective than MET in reducing risk of heart failure (OR: 0.75, 95% CI 0.58–0.95). However, comparisons between SGLT2i and INS, MET and TZD, and SU and TZD did not yielded statistically significant results. Sensitivity analyses were consistent with the original analysis (Additional file 6: Figure S2 and Additional file 7: Figure S3).

## Discussion

In our previous work, we have found that novel classes of anti-diabetic agentsshowed favorable CV safety profile in terms of MACE and all-cause mortality, despite small differences between individuals [22]. In the current study, we further explored the impact of these anti-diabetics on one specific CV outcome, namely, heart failure. Compared with prior analyses, the evidence-based hierarchies generated from this network meta-analysis were relatively similar in some parts and notably different in others.

It has been well established that TZDs, when compared with placebo, was associated with an increased risk of heart failure among diabetic patients [28–31]. In our network, TZDs consistently fared worst in both rankograms and pairwise comparisons among existent classes of anti-diabetic medications. In other words, our study once again corroborated the inimical potential of TZDs towards the precipitation or exacerbation of heart failure, strengthening the recommendation against their use in congestive heart failure [6]. On the other hand, SGLT-2 inhibitors, which ranked lowest in the risk of MACE and all-cause mortality from our previous analysis [22], were also shown to be the safest for heart failure endpoints in the current study, even in the exploratory analysis. These results were undoubtedly driven, in a large part, by the recent EMPA-REG OUTCOME, CANVAS and DECLARE trials [32–34], and were consistent with a recent systematic review and meta-analysis [35]. Preclinical studies have already alluded to the fact that sodium-glucose linked co-transporter itself is mechanistically involved for cardiac glucose uptake and energy metabolism in the heart failure setting [36, 37]. Other potential mechanisms of benefit, for instance, alleviated oxidative stress, improved arterial compliance and so on, have also been postulated [38, 39]. In one recent real-world population-based cohort study, the protective effect of SGLT-2 inhibitors against hospitalization for heart failure was observed as early as 30 days after initiation in patients with established CV diseases [40]. Currently, the efficacy of SGLT-2 inhibitors in treating congestive heart failure are being tested in outcome trials in heart failure patients regardless of concomitant diabetes.

DPP-4 inhibitors and GLP-1 agonists, or collectively termed incretin-based therapies, were found in our network to be respectively taking up 5th and 7th places in rankograms and were both statistically significantly inferior to SGLT-2 inhibitors in pairwise comparisons. For GLP-1 agonists, after LEADER and SUSTAIN-6 trials having demonstrated a reduction in CV mortality but not heart failure hospitalization [41, 42], subsequent FIGHT and LIVE trials also failed to show improvement in heart failure endpoints [43, 44]. Even though this class of drugs possesses overall cardiovascular safety [45], controversy for their use in heart failure population remains and caution is strongly advised [46]. As for DDP-4 inhibitors, the signal of harm was first identified in SAVOR-TIMI 53 and EXAMINE trials [47, 48], in which heart failure hospitalization was increased with the use of saxagliptin and alogliptin. Furthermore, in several meta-analyses and observational studies, DPP-4 inhibitors were reported to be associated with an increased risk of worsening heart failure [49–51], besides acute pancreatitis and hypoglycemia [52]. Recently, the hypothesis of adrenergically mediated cardiotoxicity mediated by DPP-4 inhibition has been proposed [53]. Experimental studies discovered that DDP-4 inhibition potentiates not only GLP-1, but also stromal cell-derived factor 1 (SDF-1), neuropeptide Y, and substance P and so on, bringing out central and peripheral sympathetic overactivity via beta-adrenergic stimulation, thereby effecting changes in cardiac inflammation, fibrosis as well as myocardial apoptosis and loss of viability [54–62]. In the SAVOR-TIMI 53 trial, the increase in heart failure hospitalization in patients treated with saxagliptin was significantly attenuated with the concomitant use of beta blockers [63], lending additional proof to the plausibility of such theory. However, in the recent CARMELINA trial, linagliptin did not seem to affect heart failure-related endpoints [64–66]. In a related commentary, one possible explanation was proposed that the unexpected findings from SAVOR TIMI-53 was due to chance, and that the overall CV effects of DPP-4 inhibitors (including heart failure) were likely neutral [67].

The complex signaling and mechanisms through which generalized insulin resistance contribute to myocardial dysfunction and ultimately heart failure has recently been reviewed by Riehle et al. [68]. Packer further proposed the potentiation of insulin signaling being instrumental in the precipitation of heart failure among patients with diabetes [69]. Specifically, direct pharmacologic effects on hyperinsulinemia of anti-diabetic medications appear to be consistently linked to the risk they confer on heart failure. In other words, medications that potentiate insulin via insulin signaling (i.e. sulfonylureas, incretin-based therapies, TZDs) tend to increase the risk of heart failure, whereas medications that mitigate hyperinsulinemia and do not act through insulin signaling (i.e., metformin and SGLT-2 inhibitors) tend to improve on heart failure outcomes [69]. In our network, SGLT-2 inhibitors and Metformin ranked comparably safest on top, whereas sulfonylureas, DDP-4 inhibitors, GLP-1 agonists and TZDs were associated with relatively increasing risk of heart failure. Thus, these results fit squarely into Packer’s conceptual framework, and further support that amplified insulin signaling may have played a major role in the clinical progression of heart failure in diabetic patients. Future studies are definitely warranted to further explore this phenomenon.

There are several limitations to our current study. First of all, the heterogeneity of trial designs and reporting styles of the included studies, as well as modest event rates in general cannot be overlooked. Therefore, these results were primarily hypothesis-generating in nature and should be interpreted with discretion. Second, information regarding heart failure being that which was with reduced or preserved ejection fraction was virtually indistinguishable in the majority of studies, preventing a deeper layer of exploration in the network which differentiates heart failure clinical phenotypes. Third, since patient-level data was inaccessible to us, other possible variables correlated with heart failure were unable to be accounted for in the current analysis. Ongoing preclinical studies and clinical trials would shed light on how these questions can be answered.

## Conclusion

Our network meta-analyses demonstrated that, in terms of heart failure risk, SGLT-2 inhibitors were the most favorable option among all classes of anti-diabetic medications; DDP-4 inhibitors and GLP-1 agonists seemed inferior to SGLT-2 inhibitors and metformin. Results from this study indicate that heart failure as a phenotypically distinct entity among diabetic complications may be significantly influenced by choice of glucose-lowering medications, and also call for future studies to further explore the dynamic interplay between pharmacologic effects of different agents, pathognomonic mechanisms of diabetes-related cardiotoxicity, and clinical progression of heart failure.

## Additional files

**Additional file 1: Table S1.** Search strategy

**Additional file 2: Table S2.** Novel anti-diabetics and recommended dosage.

**Additional file 3: Figure S1.** Flow chart of study inclusion.

**Additional file 4: Table S3.** Baseline characteristics of included studies. **Table S4.** Methodological quality assessment of included studies. **Table S5.** Heart failure events of included studies.

**Additional file 5: Table S6.** Potential inconsistency between direct and indirect comparisons assessed by the node-splitting method.

**Additional file 6: Figure S2.** Sensitivity analysis after excluding studies with an arm of fewer than 100 patients.

**Additional file 7: Figure S3.** Sensitivity analysis after excluding studies with a follow-up period of fewer than 48 weeks.

## References

[1] Kannel WB, Hjortland M, Castelli WP (1974). Role of diabetes in congestive heart failure: the Framingham study. Am J Cardiol.

[2] Aguilar D, Solomon SD, Kober L, Rouleau JL, Skali H, McMurray JJ (2004). Newly diagnosed and previously known diabetes mellitus and 1-year outcomes of acute myocardial infarction: the VALsartan In Acute myocardial iNfarcTion (VALIANT) trial. Circulation.

[3] MacDonald MR, Petrie MC, Varyani F, Ostergren J, Michelson EL, Young JB (2008). Impact of diabetes on outcomes in patients with low and preserved ejection fraction heart failure: an analysis of the Candesartan in Heart failure: assessment of Reduction in Mortality and morbidity (CHARM) programme. Eur Heart J.

[4] Pfeffer MA, Braunwald E, Moye LA, Basta L, Brown EJ, Cuddy TE (1992). Effect of captopril on mortality and morbidity in patients with left ventricular dysfunction after myocardial infarction. Results of the survival and ventricular enlargement trial. The SAVE Investigators. N Engl J Med.

[5] Solomon SD, St John Sutton M, Lamas GA, Plappert T, Rouleau JL, Skali H (2002). Ventricular remodeling does not accompany the development of heart failure in diabetic patients after myocardial infarction. Circulation.

[6] American Diabetes A (2018). 9. Cardiovascular disease and risk management: standards of medical care in diabetes-2018. Diabetes Care.

[7] Bell DS (2003). Heart failure: the frequent, forgotten, and often fatal complication of diabetes. Diabetes Care.

[8] Gilbert RE, Krum H (2015). Heart failure in diabetes: effects of anti-hyperglycaemic drug therapy. Lancet.

[9] Control G, Turnbull FM, Abraira C, Anderson RJ, Byington RP, Chalmers JP (2009). Intensive glucose control and macrovascular outcomes in type 2 diabetes. Diabetologia.

[10] Eshaghian S, Horwich TB, Fonarow GC (2006). An unexpected inverse relationship between HbA1c levels and mortality in patients with diabetes and advanced systolic heart failure. Am Heart J.

[11] Maru S, Koch GG, Stender M, Clark D, Gibowski L, Petri H (2005). Antidiabetic drugs and heart failure risk in patients with type 2 diabetes in the U.K. primary care setting. Diabetes Care.

[12] Tomova GS, Nimbal V, Horwich TB (2012). Relation between hemoglobin a(1c) and outcomes in heart failure patients with and without diabetes mellitus. Am J Cardiol.

[13] Udell JA, Cavender MA, Bhatt DL, Chatterjee S, Farkouh ME, Scirica BM (2015). Glucose-lowering drugs or strategies and cardiovascular outcomes in patients with or at risk for type 2 diabetes: a meta-analysis of randomised controlled trials. Lancet Diabetes Endocrinol.

[14] Kappel BA, Marx N, Federici M (2015). Oral hypoglycemic agents and the heart failure conundrum: lessons from and for outcome trials. Nutr Metab Cardiovasc Dis.

[15] Lehrke M, Marx N (2017). Diabetes Mellitus and Heart Failure. Am J Med.

[16] Ida S, Kaneko R, Murata K (2018). Effects of oral antidiabetic drugs on left ventricular mass in patients with type 2 diabetes mellitus: a network meta-analysis. Cardiovasc Diabetol.

[17] Tanaka A, Node K (2018). Exploration of the clinical benefits of sodium glucose co-transporter 2 inhibitors in diabetic patients with concomitant heart failure. Cardiovasc Diabetol.

[18] Dawwas GK, Smith SM, Park H (2018). Risk of heart failure hospitalization among users of dipeptidyl peptidase-4 inhibitors compared to glucagon-like peptide-1 receptor agonists. Cardiovasc Diabetol.

[19] Wu S, Cipriani A, Yang Z, Yang J, Cai T, Xu Y (2018). The cardiovascular effect of incretin-based therapies among type 2 diabetes: a systematic review and network meta-analysis. Expert Opin Drug Saf.

[20] Zheng SL, Roddick AJ, Aghar-Jaffar R, Shun-Shin MJ, Francis D, Oliver N (2018). association between use of sodium-glucose cotransporter 2 inhibitors, glucagon-like peptide 1 agonists, and dipeptidyl peptidase 4 inhibitors with all-cause mortality in patients with type 2 diabetes: a systematic review and meta-analysis. JAMA.

[21] Alfayez OM, Al Yami MS, Alshibani M, Fallatah SB, Al Khushaym NM, Alsheikh R (2019). Network meta-analysis of nine large cardiovascular outcome trials of new antidiabetic drugs. Prim Care Diabetes.

[22] Zhuang XD, He X, Yang DY, Guo Y, He JG, Xiao HP (2018). Comparative cardiovascular outcomes in the era of novel anti-diabetic agents: a comprehensive network meta-analysis of 166,371 participants from 170 randomized controlled trials. Cardiovasc Diabetol.

[23] Moher D, Liberati A, Tetzlaff J, Altman DG, Group P (2009). Preferred reporting items for systematic reviews and meta-analyses: the PRISMA statement. J Clin Epidemiol.

[24] Caldwell DM, Ades AE, Higgins JP (2005). Simultaneous comparison of multiple treatments: combining direct and indirect evidence. BMJ.

[25] Green S HJ. Cochrane handbook for systematic reviews of interventions. 2008.

[26] Salanti G, Ades AE, Ioannidis JP (2011). Graphical methods and numerical summaries for presenting results from multiple-treatment meta-analysis: an overview and tutorial. J Clin Epidemiol.

[27] Veroniki AA, Vasiliadis HS, Higgins JP, Salanti G (2013). Evaluation of inconsistency in networks of interventions. Int J Epidemiol.

[28] Hernandez AV, Usmani A, Rajamanickam A, Moheet A (2011). Thiazolidinediones and risk of heart failure in patients with or at high risk of type 2 diabetes mellitus: a meta-analysis and meta-regression analysis of placebo-controlled randomized clinical trials. Am J Cardiovasc Drugs.

[29] Lago RM, Singh PP, Nesto RW (2007). Congestive heart failure and cardiovascular death in patients with prediabetes and type 2 diabetes given thiazolidinediones: a meta-analysis of randomised clinical trials. Lancet.

[30] Singh S, Loke YK, Furberg CD (2007). Thiazolidinediones and heart failure: a teleo-analysis. Diabetes Care.

[31] de Jong M, van der Worp HB, van der Graaf Y, Visseren FLJ, Westerink J (2017). Pioglitazone and the secondary prevention of cardiovascular disease. A meta-analysis of randomized-controlled trials. Cardiovasc Diabetol.

[32] Neal B, Perkovic V, Matthews DR (2017). Canagliflozin and cardiovascular and renal events in type 2 diabetes. N Engl J Med.

[33] Wiviott SD, Raz I, Bonaca MP, Mosenzon O, Kato ET, Cahn A (2019). Dapagliflozin and cardiovascular outcomes in type 2 diabetes. N Engl J Med.

[34] Zinman B, Wanner C, Lachin JM, Fitchett D, Bluhmki E, Hantel S (2015). Empagliflozin, cardiovascular outcomes, and mortality in type 2 diabetes. N Engl J Med.

[35] Zelniker TA, Wiviott SD, Raz I, Im K, Goodrich EL, Bonaca MP (2019). SGLT2 inhibitors for primary and secondary prevention of cardiovascular and renal outcomes in type 2 diabetes: a systematic review and meta-analysis of cardiovascular outcome trials. Lancet.

[36] Banerjee SK, McGaffin KR, Pastor-Soler NM, Ahmad F (2009). SGLT1 is a novel cardiac glucose transporter that is perturbed in disease states. Cardiovasc Res.

[37] Banerjee SK, Wang DW, Alzamora R, Huang XN, Pastor-Soler NM, Hallows KR (2010). SGLT1, a novel cardiac glucose transporter, mediates increased glucose uptake in PRKAG2 cardiomyopathy. J Mol Cell Cardiol.

[38] Ferrannini E, Mark M, Mayoux E (2016). CV protection in the EMPA-REG OUTCOME Trial: a “thrifty substrate” hypothesis. Diabetes Care.

[39] Marx N, McGuire DK (2016). Sodium-glucose cotransporter-2 inhibition for the reduction of cardiovascular events in high-risk patients with diabetes mellitus. Eur Heart J.

[40] Kim YG, Han SJ, Kim DJ, Lee KW, Kim HJ (2018). Association between sodium glucose co-transporter 2 inhibitors and a reduced risk of heart failure in patients with type 2 diabetes mellitus: a real-world nationwide population-based cohort study. Cardiovasc Diabetol.

[41] Marso SP, Bain SC, Consoli A, Eliaschewitz FG, Jodar E, Leiter LA (2016). Semaglutide and cardiovascular outcomes in patients with type 2 diabetes. N Engl J Med.

[42] Marso SP, Daniels GH, Brown-Frandsen K, Kristensen P, Mann JF, Nauck MA (2016). Liraglutide and cardiovascular outcomes in type 2 diabetes. N Engl J Med.

[43] Jorsal A, Kistorp C, Holmager P, Tougaard RS, Nielsen R, Hanselmann A (2017). Effect of liraglutide, a glucagon-like peptide-1 analogue, on left ventricular function in stable chronic heart failure patients with and without diabetes (LIVE)-a multicentre, double-blind, randomised, placebo-controlled trial. Eur J Heart Fail.

[44] Margulies KB, Hernandez AF, Redfield MM, Givertz MM, Oliveira GH, Cole R (2016). Effects of liraglutide on clinical stability among patients with advanced heart failure and reduced ejection fraction: a randomized clinical trial. JAMA.

[45] Bethel MA, Patel RA, Merrill P, Lokhnygina Y, Buse JB, Mentz RJ (2018). Cardiovascular outcomes with glucagon-like peptide-1 receptor agonists in patients with type 2 diabetes: a meta-analysis. Lancet Diabetes Endocrinol.

[46] Scheen AJ (2017). GLP-1 receptor agonists and heart failure in diabetes. Diabetes Metab.

[47] Scirica BM, Bhatt DL, Braunwald E, Steg PG, Davidson J, Hirshberg B (2013). Saxagliptin and cardiovascular outcomes in patients with type 2 diabetes mellitus. N Engl J Med.

[48] White WB, Cannon CP, Heller SR, Nissen SE, Bergenstal RM, Bakris GL (2013). Alogliptin after acute coronary syndrome in patients with type 2 diabetes. N Engl J Med.

[49] Clifton P (2014). Do dipeptidyl peptidase IV (DPP-IV) inhibitors cause heart failure?. Clin Ther.

[50] Li L, Li S, Deng K, Liu J, Vandvik PO, Zhao P (2016). Dipeptidyl peptidase-4 inhibitors and risk of heart failure in type 2 diabetes: systematic review and meta-analysis of randomised and observational studies. BMJ.

[51] Monami M, Dicembrini I, Mannucci E (2014). Dipeptidyl peptidase-4 inhibitors and heart failure: a meta-analysis of randomized clinical trials. Nutr Metab Cardiovasc Dis.

[52] Zhang Z, Chen X, Lu P, Zhang J, Xu Y, He W (2017). Incretin-based agents in type 2 diabetic patients at cardiovascular risk: compare the effect of GLP-1 agonists and DPP-4 inhibitors on cardiovascular and pancreatic outcomes. Cardiovasc Diabetol.

[53] Packer M (2018). Do DPP-4 inhibitors cause heart failure events by promoting adrenergically mediated cardiotoxicity? Clues from laboratory models and clinical trials. Circ Res.

[54] Boschmann M, Engeli S, Dobberstein K, Budziarek P, Strauss A, Boehnke J (2009). Dipeptidyl-peptidase-IV inhibition augments postprandial lipid mobilization and oxidation in type 2 diabetic patients. J Clin Endocrinol Metab.

[55] Ceholski DK, Turnbull IC, Pothula V, Lecce L, Jarrah AA, Kho C (2017). CXCR4 and CXCR7 play distinct roles in cardiac lineage specification and pharmacologic beta-adrenergic response. Stem Cell Res.

[56] Dehlin HM, Manteufel EJ, Monroe AL, Reimer MH, Levick SP (2013). Substance P acting via the neurokinin-1 receptor regulates adverse myocardial remodeling in a rat model of hypertension. Int J Cardiol.

[57] Jackson EK, Dubinion JH, Mi Z (2008). Effects of dipeptidyl peptidase iv inhibition on arterial blood pressure. Clin Exp Pharmacol Physiol.

[58] Jackson EK, Mi Z (2008). Sitagliptin augments sympathetic enhancement of the renovascular effects of angiotensin II in genetic hypertension. Hypertension.

[59] Luo G, Xu X, Guo W, Luo C, Wang H, Meng X (2015). Neuropeptide Y damages the integrity of mitochondrial structure and disrupts energy metabolism in cultured neonatal rat cardiomyocytes. Peptides.

[60] Marney A, Kunchakarra S, Byrne L, Brown NJ (2010). Interactive hemodynamic effects of dipeptidyl peptidase-IV inhibition and angiotensin-converting enzyme inhibition in humans. Hypertension.

[61] Robinson P, Kasembeli M, Bharadwaj U, Engineer N, Eckols KT, Tweardy DJ (2016). Substance P receptor signaling mediates doxorubicin-induced cardiomyocyte apoptosis and triple-negative breast cancer chemoresistance. Biomed Res Int.

[62] Zhu X, Gillespie DG, Jackson EK (2015). NPY1-36 and PYY1-36 activate cardiac fibroblasts: an effect enhanced by genetic hypertension and inhibition of dipeptidyl peptidase 4. Am J Physiol Heart Circ Physiol.

[63] Scirica BM, Braunwald E, Raz I, Cavender MA, Morrow DA, Jarolim P (2015). Heart failure, saxagliptin, and diabetes mellitus: observations from the SAVOR-TIMI 53 randomized trial. Circulation.

[64] Rosenstock J, Perkovic V, Alexander JH, Cooper ME, Marx N, Pencina MJ (2018). Rationale, design, and baseline characteristics of the CArdiovascular safety and Renal Microvascular outcomE study with LINAgliptin (CARMELINA((R))): a randomized, double-blind, placebo-controlled clinical trial in patients with type 2 diabetes and high cardio-renal risk. Cardiovasc Diabetol.

[65] Rosenstock J, Perkovic V, Johansen OE, Cooper ME, Kahn SE, Marx N (2019). Effect of linagliptin vs placebo on major cardiovascular events in adults with type 2 diabetes and high cardiovascular and renal risk: the CARMELINA randomized clinical trial. JAMA.

[66] McGuire DK, Alexander JH, Johansen OE, Perkovic V, Rosenstock J, Cooper ME (2019). Linagliptin effects on heart failure and related outcomes in individuals with type 2 diabetes mellitus at high cardiovascular and renal risk in CARMELINA. Circulation.

[67] Zannad F, Rossignol P (2019). Dipeptidyl peptidase-4 inhibitors and the risk of heart failure. Circulation.

[68] Riehle C, Abel ED (2016). Insulin signaling and heart failure. Circ Res.

[69] Packer M (2018). Potentiation of insulin signaling contributes to heart failure in type 2 diabetes: a hypothesis supported by both mechanistic studies and clinical trials. JACC Basic Transl Sci.

