# Supplementary material for: Comparative outcomes of heart failure among existent classes of anti-diabetic agents: a network meta-analysis of 171,253 participants from 91 randomized controlled trials
Source: Cardiovasc Diabetol. 2019 Apr 8;18:47. doi: 10.1186/s12933-019-0853-x (PMC6454617; doi:10.1186/s12933-019-0853-x)
Supplement: Supplementary file 1 — Additional file 1: Table S1. Search strategy [file 12933_2019_853_MOESM1_ESM.docx]

| **MEDLINE** | **Embase** |
| --- | --- |
| 1. exp Diabetes Mellitus Type 2/ 2. diabetes mellitus/ 3. non-insulin dependent diabetes mellitus 4. ((diabetes or diabetes mellitus or diabetic*) adj1 (type 2 or type ii or type ii or non-insulin dependent or noninsulin dependent or adult onset or mature onset or late onset)).tw) 5. NIDDD.tw. 6. or/1-4 7. glucagon like peptide 1 receptor agonist/ 8. (glp-1 or glp-1 receptor inhibitor* or glp-1 agonist*).tw. 9. albiglutide/ 10. dulaglutide/ 11. liraglutide/ 12. lixisenatide/ 13. albiglutide.tw. 14. dulaglutide.tw. 15. liraglutide.tw. 16. lixisenatide.tw. 17. dipeptidyl peptidase iv inhibitor/ 18. (dpp4 or dpp 4 or dpp iv).tw. 19. alogliptin/ 20. saxagliptin/ 21. sitagliptin/ 22. vildagliptin/ 23. alogliptin.tw. 24. saxagliptin.tw. 25. sitagliptin.tw. 26. vildagliptin.tw. 27. sodium glucose cotransporter 2 inhibitor/ 28. (sodium glucose cotransporter 2 inhibitor* or sodium glucose cotransporter ii inhibitor or sglt 2 inhibitor*).tw. 29. canagliflozin/ 30. dapagliflozin/ 31. empagliflozin/ 32. canagliflozin.tw. 33. dapagliflozin.tw. 34. empagliflozin.tw. 35. antidiabetic agent/ 36. oral antidiabetic agent/ 37. exp insulin, Long acting/ 38. ((long acting or longer acting or intermediate acting) adj insulin*).tw. 39. insulin degludec/ 40. insulin detemir/ 41. insulin glargine/ 42. insulin zinc suspension 43. insulin aspart/ 44. insulin lispro/ 45. isophane insulin/ 46. meglitinide/ 47. mitiglinide/ 48. nateglinide/ 49. repaglinide/ 50. amylin derivative/ 51. pramlintide/ 52. biguanide derivative/ 53. metformin.tw. 54. sulphonylurea*.tw. 55. acetohexamide/ 56. carbutamide/ 57. chlorpropamide/ 58. glibornuride/ 59. glibenclamide/ 60. gliclazide/ 61. glimepiride.tw. 62. glipizide/ 63. gliquidone/ 64. tolazamide/ 65. acetohexamide.tw. 66. carbutamide.tw. 67. chlorpropamide.tw. 68. glibenclamide.tw. 69. gliclazide.tw. 70. glyburide.tw. 71. glitazone derivative/ 72. thiazolidinedione*.tw. 73. pioglitazone/ 74. rivoglitazone/ 75. rosiglitazone/ 76. pioglitazone.tw. 77. rivoglitazone.tw. 78. rosiglitazone.tw. 79. or/6-77 80. and/5,78 81. randomized controlled trial.pt. 82. controlled clinical trial.pt. 83. pragmatic clinical trial.pt. 84. double-blind.ab. 85. single-blind.ab. 86. randomized.ab. 87. placebo.ab. 88. trial.ti. 89. or/81-88 90. animals/not(humans/and animal/) 91. 89 not 90 | 1. diabetes mellitus/ 2. non-insulin dependent diabetes mellitus 3. ((diabetes or diabetes mellitus or diabetic*) adj1 (type 2 or type ii or type ii or non-insulin dependent or noninsulin dependent or adult onset or mature onset or late onset)).tw) 4. NIDDD.tw. 5. or/1-4 6. glucagon like peptide 1 receptor agonist/ 7. (glp-1 or glp-1 receptor inhibitor* or glp-1 agonist*).tw. 8. albiglutide/ 9. dulaglutide/ 10. liraglutide/ 11. lixisenatide/ 12. albiglutide.tw. 13. dulaglutide.tw. 14. liraglutide.tw. 15. lixisenatide.tw. 16. dipeptidyl peptidase iv inhibitor/ 17. (dpp4 or dpp 4 or dpp iv).tw. 18. alogliptin/ 19. saxagliptin/ 20. sitagliptin/ 21. vildagliptin/ 22. alogliptin.tw. 23. saxagliptin.tw. 24. sitagliptin.tw. 25. vildagliptin.tw. 26. sodium glucose cotransporter 2 inhibitor/ 27. (sodium glucose cotransporter 2 inhibitor* or sodium glucose cotransporter ii inhibitor or sglt 2 inhibitor*).tw. 28. canagliflozin/ 29. dapagliflozin/ 30. empagliflozin/ 31. canagliflozin.tw. 32. dapagliflozin.tw. 33. empagliflozin.tw. 34. antidiabetic agent/ 35. oral antidiabetic agent/ 36. long acting insulin/ 37. ((long acting or longer acting or intermediate acting) adj insulin*).tw. 38. insulin degludec/ 39. insulin detemir/ 40. insulin glargine/ 41. insulin zinc suspension 42. insulin aspart/ 43. insulin lispro/ 44. isophane insulin/ 45. meglitinide/ 46. mitiglinide/ 47. nateglinide/ 48. repaglinide/ 49. amylin derivative/ 50. pramlintide/ 51. biguanide derivative/ 52. metformin.tw. 53. sulphonylurea*.tw. 54. acetohexamide/ 55. carbutamide/ 56. chlorpropamide/ 57. glibornuride/ 58. glibenclamide/ 59. gliclazide/ 60. glimepiride.tw. 61. glipizide/ 62. gliquidone/ 63. tolazamide/ 64. acetohexamide.tw. 65. carbutamide.tw. 66. chlorpropamide.tw. 67. glibenclamide.tw. 68. gliclazide.tw. 69. glyburide.tw. 70. glitazone derivative/ 71. thiazolidinedione*.tw. 72. pioglitazone/ 73. rivoglitazone/ 74. rosiglitazone/ 75. pioglitazone.tw. 76. rivoglitazone.tw. 77. rosiglitazone.tw. 78. or/6-77 79. and/5,78 80. randomized controlled trial/ 81. double-blind/ 82. single-blind/ 83. random$.tw. 84. factorial$.tw. 85. assign$.tw. 86. allocate$.tw. 87. or/80-86 88. and/79,87 89. MEDLINE.cr 90. 88 not 89 91. (mouse or mice or murine or rat or rats or dog or animal*).ti. 92. 90 not 91 |
