# Supplementary material for: Comparative outcomes of heart failure among existent classes of anti-diabetic agents: a network meta-analysis of 171,253 participants from 91 randomized controlled trials
Source: Cardiovasc Diabetol. 2019 Apr 8;18:47. doi: 10.1186/s12933-019-0853-x (PMC6454617; doi:10.1186/s12933-019-0853-x)
Supplement: Supplementary file 2 — Additional file 2: Table S2. Novel anti-diabetics and recommended dosage. [file 12933_2019_853_MOESM2_ESM.docx]

| **Drugs** | **Recommended Dosage** | **Dosage in Patients with Renal Impairment** |
| --- | --- | --- |
| **DPP-4 inhibitors** | | |
| Sitagliptin  (JANUVIA) | 100 mg once daily | Mild: no dosage adjustment  Moderate: 50 mg once daily  Severe or end-stage: 25 mg once daily |
| Saxagliptin (ONGLYZA) | 2.5 mg or 5 mg once daily | Mild: no dosage adjustment  Moderate, severe or end-stage: 2.5 mg once daily |
| Vildagliptin  (Galvus^*^) | 100 mg once daily or 50mg twice daily  50 mg once daily if combined use with a sulphonylurea | Mild: no dosage adjustment  Moderate, severe or end-stage: 50 mg once daily |
| Alogliptin  (NESINA) | 25 mg once daily | Mild: no dosage adjustment  Moderate: 12.5mg once daily  Severe or end-stage: 6.25 mg once daily |
| Linagliptin (TRADJENTA) | 5 mg once daily | No dosage adjustment |
| **GLP-1 receptor agonists** | | |
| Liraglutide  (VICTOZA) | 1.2 mg once daily  1.8 mg once daily for inadequate glycemic control | No dosage adjustment |
| Dulaglutide (TRULICITY) | 0.75 mg once weekly  1.5 mg once weekly for inadequate glycemic control | No dosage adjustment |
| Albiglutide (TANZEUM) | 30 mg once weekly  50 mg once weekly for inadequate glycemic control | No dosage adjustment |
| Lixisenatide (LYXUMIA^*^) | 20 μg once daily | Mild and moderate: No dosage adjustment  Severe or end-stage: not recommended |
| Exenatide (BYDUREON) | 2mg once weekly | Moderate: use with caution  Severe or end-stage: not recommended |
| Exenatide  (BYETTA) | 5 μg bid or 10 μg bid based on clinical response | Moderate: use with caution  Severe or end-stage: not recommended |
| **SGLT-2 inhibitors** | | |
| Empagliflozin (JARDIANCE) | 10 mg once daily  25 mg once daily if JARDIANCE is tolerated | eGFR > 45 mL/min/1.73 m2: No dosage adjustment  eGFR < 45 mL/min/1.73 m2: Not recommended |
| Dapagliflozin (FARXIGA) | 5 mg once daily  10 mg once daily if FARXIGA is tolerated | eGFR > 60 mL/min/1.73 m2: No dosage adjustment  eGFR < 60 mL/min/1.73 m2: Not recommended |
| Canagliflozin (INVOKANA) | 100 mg once daily  300 mg once daily if INVOKANA is tolerated | eGFR 45~60 mL/min/1.73 m2: 100 mg once daily  eGFR < 45 mL/min/1.73 m2: Not recommended |
| ^*^ Still not approved by FDA, but already approved by the European Medicines Agency | | |
