# Supplementary material for: Comparative outcomes of heart failure among existent classes of anti-diabetic agents: a network meta-analysis of 171,253 participants from 91 randomized controlled trials
Source: Cardiovasc Diabetol. 2019 Apr 8;18:47. doi: 10.1186/s12933-019-0853-x (PMC6454617; doi:10.1186/s12933-019-0853-x)
Supplement: Supplementary file 3 — Additional file 3: Figure S1. Flow chart of study inclusion. [file 12933_2019_853_MOESM3_ESM.docx]

1,342 Excluded

1,136 No heart failure outcome

82 Not randomized trials

29 Not type 2 diabetes patients

95 Pooled analysis

91 Included

6,287 Excluded

4,272 Not RCT

1,344 No drug comparison

11 Not recommended dosage

72 Crossover trials

588 Follow-up < 24 weeks

1,433 Full-text reports retrieved

1,023 Excluded (duplicate records)

7,720 Records screened by title and abstract

8,743 Potentially relevant reports identified and screened for retrieval from electronic search

2,378 MEDLINE

1,767 EMbase

4,562 Cochrance Central Register of Randomized Trials

36 Reference lists from included studies
