# Supplementary material for: Comparative outcomes of heart failure among existent classes of anti-diabetic agents: a network meta-analysis of 171,253 participants from 91 randomized controlled trials
Source: Cardiovasc Diabetol. 2019 Apr 8;18:47. doi: 10.1186/s12933-019-0853-x (PMC6454617; doi:10.1186/s12933-019-0853-x)
Supplement: Supplementary file 4 — Additional file 4: Table S3. Baseline characteristics of included studies. Table S4. Methodological quality assessment of included studies. Table S5. Heart failure events of included studies. [file 12933_2019_853_MOESM4_ESM.docx]

| Table S3 Baseline characteristics of included studies | | | | | | | | | |
| --- | --- | --- | --- | --- | --- | --- | --- | --- | --- |
| Study | International | Centers | Special character of population | Antidabetic | Comparator | Drug used across groups | Treatment duration (weeks) | Mean age (years) | Male Patients (%) |
| NCT00968708^1,2^ | Yes | 898 | High cardiovascular risk | Alogliptin | Placebo | OAD or Insulin | 76(Mean) | 61^#^ | 67.9 |
| NCT00856284^3^ | Yes | 310 | None | Alogliptin | Glipizide | Metformin | 104 | 55.5 | 50.8 |
| NCT00707993^4^ | Yes | 110 | Age≥65 | Alogliptin | Glipizide | None | 52 | 70.0 | 44.9 |
| NCT00328627^5^ | Yes | 327 | None | Alogliptin | Pioglitazone, Placebo | Metformin | 26 | 54.7 | 44.5 |
| NCT00432276^6^ | Yes | NR | None | Alogliptin | Pioglitazone | Metformin + Pioglitazone | 52 | 55.1 | 51.6 |
| NCT00286442^7^ | Yes | 115 | None | Alogliptin | Placebo | Metformin | 26 | 55.0 | 52.2 |
| NCT00286494^8^ | Yes | 125 | None | Alogliptin | Placebo | Pioglitazone | 26 | 55.3 | 60.1 |
| NCT00800683^9^ | Yes | 53 | Severe renal impairment | Linagliptin | Placebo | OAD or Insulin | 52 | 64.4 | 60.2 |
| NCT00954447^10^ | Yes | 167 | None | Linagliptin | Placebo | Insulin ±Metformin/Pioglitazone | 52 | 60.0 | 52.1 |
| NCT00622284^11^ | Yes | 209 | None | Linagliptin | Glimepiride | Metformin | 104 | 59.8 | 60.0 |
| NCT00798161^12^ | Yes | 133 | None | Linagliptin | Metformin, Placebo | None | 24 | 54.9 | 54.7 |
| NCT01897532^13^ | Yes | 605 | High cardiovascular and renal risk | Linagliptin | Placebo | None, OAD or Insulin | 114(median) | 65.9 | 62.9 |
| NCT01006603^14^ | Yes | 152 | Age≥65 | Saxagliptin | Glimepiride | Metformin | 52 | 72.6 | 61.8 |
| NCT00757588^15,16^ | Yes | 72 | None | Saxagliptin | Placebo | Insulin ± Metformin | 52 | 57.2 | 41.3 |
| NCT00575588^17,18^ | Yes | NR | None | Saxagliptin | Glipizide | Metformin | 104 | 57.5 | 51.6 |
| NCT00121641^19,20^ | NR | NR | None | Saxagliptin | Placebo | None | 208 | 53.7 | 52.5 |
| NCT01107886^21^ | Yes | 788 | High cardiovascular risk | Saxagliptin | Placebo | OAD or Insulin | 104(Median) | 65.1 | 64.9 |
| NCT00121667^22^ | Yes | NR | None | Saxagliptin | Placebo | Metformin | 24 | 54.7 | 50.2 |
| NCT00295633^23,24^ | NR | 172 | None | Saxagliptin | Placebo | Thiazolidinedione | 76 | 54.0 | 49.6 |
| NCT00327015^25,26^ | Yes | NR | None | Saxagliptin | Placebo | Metformin | 76 | 52.5 | 51.2 |
| NCT00614939^27^ | Yes | NR | Renal impairment | Saxagliptin | Placebo | OAD or Insulin | 52 | 66.5 | 42.9 |
| NCT01289990^28,29^(1) | Yes | 124 | None | Sitagliptin, Empagliflozin | Placebo | None | 76 | 55.0 | 61.0 |
| NCT00790205^30^ | Yes | 673 | High cardiovascular risk | Sitagliptin | Placebo | OAD or Insulin | 144(Mean) | 65.5 | 70.7 |
| NCT01098539^31^ | Yes | 134 | Renal impairment | Sitagliptin, Albiglutide |  | OAD | 52 | 63.3 | 53.7 |
| NCT00509236^32^ | Yes | 31 | Severe renal impairment | Sitagliptin | Glipizide | None | 54 | 60.0 | 59.7 |
| NCT00509262^33^ | Yes | NR | Moderate to severe renal impairment | Sitagliptin | Glipizide | None | 54 | 64.5 | 57.0 |
| NCT01046110^34^ | Yes | 78 | None | Sitagliptin | Insulin degludec | OAD | 26 | 55.7 | 58.6 |
| NCT00700817^35,36^ | Yes | 158 | None | Sitagliptin, Liraglutide |  | Metformin | 52 | 55.3 | 53.0 |
| NCT00094770^37,38^ | Yes | NR | None | Sitagliptin | Glipizide | Metformin | 104 | 56.7 | 59.2 |
| NCT00395343^39^ | Yes | 100 | None | Sitagliptin | Placebo | Insulin | 24 | 57.8 | 50.9 |
| NCT01189890^40^ | Yes | 85 | Age≥65 | Sitagliptin | Glimepiride | None | 30 | 70.7 | 43.8 |
| NCT00838903^41^ | Yes | 289 | None | Sitagliptin, Albiglutide | Placebo, Glimepiride | Metformin | 156 | 54.5 | 47.6 |
| NCT00106340^42,43^ | Yes | 402 | None | Vildagliptin | Glimepiride | Metformin | 52 | 57.5 | 53.4 |
| NCT00849017^42,43^ | Yes | 143 | None | Albiglutide | Placebo | None | 156 | 52.9 | 55.1 |
| NCT00839527^45^ | Yes | 234 | None | Albiglutide | Pioglitazone, Placebo | Metformin and Glimepiride | 156 | 55.2 | 53.2 |
| NCT00849056^46^ | Yes | 158 | None | Albiglutide | Placebo | Pioglitazone ± Metformin | 156 | 55.0 | 59.8 |
| NCT00838916^47^ | Yes | 222 | None | Albiglutide | Insulin glargine | Metformin ± Sulfonylurea | 52 | 55.5 | 56.1 |
| NCT01191268^48^ | Yes | 105 | None | Dulaglutide | Insulin glargine | Metformin | 52 | 59.0 | 53.5 |
| NCT01126580^49^ | Yes | NR | None | Dulaglutide | Metformin | OAD | 52 | 44.0 | 56.0 |
| NCT01064687^50^ | Yes | NR | None | Dulaglutide, Exenatide | Placebo | None | 26 | 56.0 | 55.6 |
| NCT00359762^51,52^ | Yes | 128 | None | Exenatide | Glimepiride | Insulin glargine | 108(Mean) | 57.2 | 53.8 |
| NCT00960661^53^ | Yes | 108 | None | Exenatide | Insulin lispro | None | 30 | 59.7 | 48.0 |
| NCT00935532^54^ | Yes | NR | None | Exenatide | Insulin glargine | Metformin + Sulfonylurea | 26 | 56.8 | 67.9 |
| NCT01179048^55^ | Yes | 410 | High cardiovascular risk | Liraglutide | Placebo | OAD | 182(Median) | 64.3 | 64.3 |
| NCT00294723^56,57,58^ | Yes | 138 | None | Liraglutide | Glimepiride | Insulin degludec + Metformin | 104 | 53.0 | 50.0 |
| NCT00318461^59^ | Yes | 170 | None | Liraglutide | Placebo, Glimepiride | None | 26 | 57.0 | 57.0 |
| NCT01147250^60^ | Yes | NR | High cardiovascular risk | Lixisenatide | Placebo | None or OAD | 100(Median) | 60.3 | 69.3 |
| NCT00975286^61^ | Yes | 140 | None | Lixisenatide | Placebo | Insulin ± Metformin | 24 | 56.0 | 50.0 |
| NCT01106651^62,63^ | Yes | 90 | Age≥55 | Canagliflozin | Placebo | Metformin | 104 | 63.6 | 55.5 |
| NCT00968812^64,65^ | Yes | 157 | None | Canagliflozin | Glimepiride | None, OAD or Insulin | 104 | 56.2 | 52.0 |
| NCT01064414^66,67^ | NR | NR | Renal impairment | Canagliflozin | Placebo | None or OAD | 52 | 68.5 | 60.6 |
| NCT01031680^68^ | Yes | NR | High cardiovascular risk | Dapagliflozin | Placebo | None | 52 | 62.9 | 68.3 |
| NCT00660907^69,70,71^ | Yes | 95 | None | Dapagliflozin | Glipizide | OAD or Insulin | 52 | 59.0 | 55.1 |
| NCT00663260^72^ | Yes | 111 | Renal impairment | Dapagliflozin | Placebo | Sitagliptin ± Metformin | 104 | 67.0 | 55.0 |
| NCT01042977^73^ | Yes | 173 | High cardiovascular risk | Dapagliflozin | Placebo | None, OAD or Insulin | 52 | 63.7 | 67.0 |
| NCT00683878^74^ | Yes | 105 | None | Dapagliflozin | Placebo | Metformin | 48 | 53.5 | 49.5 |
| NCT00680745^75^ | Yes | 84 | None | Dapagliflozin | Placebo | Pioglitazone | 24 | 59.8 | 52.0 |
| NCT01730534^76^ | Yes | 882 | High cardiovascular risk | Dapagliflozin | Placebo | None, OAD or Insulin | 218(median) | 64.0 | 62.6 |
| NCT01131676^77^ | Yes | 590 | High cardiovascular risk | Empagliflozin | Placebo | Pioglitazone and Metformin | 135(Median) | 63.1 | 71.4 |
| NCT01368081^78^ | No(Japan) | 86 | None | Empagliflozin | Metformin | None or Pioglitazone ± Metformin | 52 | 61.2 | 71.4 |
| NCT01164501^79^ | Yes | 127 | Renal impairment | Empagliflozin | Placebo | Sulfonylurea | 52 | 64.7 | 57.8 |
| NCT01289990^28,29^（2）^*^ | Yes | 148 | None | Empagliflozin | Placebo | OAD or Insulin | 76 | 56.4 | 53.7 |
| NCT01167881^80^ | Yes | 173 | None | Empagliflozin | Glimepiride | None or OAD | 104 | 56.0 | 55.0 |
| NCT01306214^81^ | Yes | 104 | None | Empagliflozin | Placebo | Metformin | 52 | 56.7 | 45.0 |
| NCT00521742^82^ | No(USA) | NR | Early heart failure | Glyburide | Pioglitazone | None | 52 | 64 | 56 |
| 1998UKPDS^83^ | No(UK) | 23 | None | Glibenclamide | Insulin | None | 520 | 54 | 62.2 |
| 2008Hamann^84^ | Yes | 118 | None | Glibenclamide or Gliclazide | Rosiglitazone | Metformin | 52 | 58.9 | 52 |
| NCT00513630^85^ | No(China) | 15 | Coronary heart disease | Glipizide | Metformin | None | 260 | 63.3 | 77.6 |
| NCT00484198^86^ | Yes | 254 | None | Pioglitazone | Placebo | None or OAD | 26 | 55.1 | 52.4 |
| NCT00116831^87^ | Yes | 92 | Coronary heart disease | Glipizide | Rosiglitazone | None | 78 | 61.0 | 67.9 |
| NCT00494312^88^ | No(USA) | 171 | None | Glibenclamide | Pioglitazone | None or Metformin | 144 | 54^#^ | 56.4 |
| NCT00225277^89^ | Yes | 97 | Coronary heart disease | Glimepiride | Pioglitazone | None | 156 | 59.8 | 67.4 |
| 2006Jain^90^ | No(USA) | 65 | None | Glyburide | Pioglitazone | None | 56 | 52.1 | 54.6 |
| NCT00279045^91^ | Yes | 488 | None | Glyburide, Rosiglitazone | Metformin | None | 408(median) | 56.9 | 57.7 |
| 2005Charbonnel^91^ | Yes | 91 | None | Pioglitazone | Metformin | Sulfonylurea | 104 | 60 | 54.1 |
| NCT00099853^93^ | Yes | 123 | None | Vildagliptin | Placebo | Pioglitazone | 24 | 54.4 | 41.6 |
| 2007Home^94^ | Yes | 338 | None | Rosiglitazone | Metformin, Sulfonylurea | Metformin or Sulfonylurea | 195 | 58.5 | 51.6 |
| 2006Mazzone | No(USA) | 28 | None | Pioglitazone | Glimepiride | None, OAD or Insulin | 72 | 59.6 | 63.1 |
| NCT00770653^96^ | NA | NA | None | Pioglitazone | Glimepiride | Metformin | 24 | 59 | 64.9 |
| 2008Chou^97^ | Yes | 155 | None | Rosiglitazone | Glimepiride | None | 28 | 53.3 | 58.9 |
| 2005Matthews^98^ | Yes | 75 | None | Pioglitazone | Gliclazide | Metformin | 52 | 56.5 | 50.0 |
| 2002St John Sutton^99^ | No(USA) | 19 | None | Rosiglitazone | Glyburide | None | 52 | 55.6 | 72.9 |
| NCT01646320^100^ | NA | NA | None | Dapagliflozin | Placebo | Saxagliptin+Metformin | 52 | 55.1 | 45.6 |
| NCT01907854^101^ | Yes | 86 | None | Liraglutide | Sitagliptin | Metformin | 26 | 56.4 | 60 |
| NCT02058147^102^ | NA | NA | None | Liraglutide | Insulin | Metformin | 30 | 58.4 | 52.8 |
| NCT01768559^103^ | Yes | 199 | None | Lixisenatide | Insulin | Insulin±Metformin | 26 | 59.8 | 45.3 |
| NCT01032629 and NCT01989754^104^ | Yes | 667 | High cardiovascular risk | Canagliflozin | Placebo | None, OAD or Insulin | 188(mean) | 63.3 | 64.2 |
| NCT01792518^105^ | Yes | 80 | None | Linagliptin | Placebo | None, OAD or Insulin | 24 | 60.6 | 63.6 |
| NCT01144338^106^ | Yes | 687 | None | Exenatide | Placebo | None, OAD or Insulin | 166(mean) | 62.0 | 62.0 |
| NCT02229383^107^ | Yes | 126 | None | Exenatide | Placebo | Insulin±Metformin | 28 | 57.7 | 47.9 |
| ^*^ Baseline information was only available for a part of the population; ^#^ Mean was estimated by median; ± with or without | | | | | | | | | |

| Table S4 Methodological quality assessment of included studies | | | | | | | |
| --- | --- | --- | --- | --- | --- | --- | --- |
| Study | Random sequence generation | Allocation concealment | Blinding of participants and personnel | Blinding of outcome assessment | Incomplete data | Selective reporting | Other source of bias |
| NCT00968708^1,2^ | Unknown | Unknown | Low risk | Low risk | Low risk | Low risk | Low risk |
| NCT00856284^3^ | Unknown | Unknown | Low risk | Low risk | Low risk | Low risk | High risk |
| NCT00707993^4^ | Unknown | Unknown | Low risk | High risk | Low risk | Low risk | High risk |
| NCT00328627^5^ | Unknown | Unknown | Low risk | High risk | Low risk | Low risk | Low risk |
| NCT00432276^6^ | Unknown | Unknown | Low risk | High risk | Low risk | Low risk | High risk |
| NCT00286442^7^ | Low risk | Low risk | Low risk | High risk | Low risk | Low risk | Low risk |
| NCT00286494^8^ | Low risk | Low risk | Low risk | High risk | Low risk | Low risk | Low risk |
| NCT00800683^9^ | Unknown | Unknown | Low risk | Low risk | Low risk | Low risk | Low risk |
| NCT00954447^10^ | Low risk | Low risk | Low risk | Low risk | Low risk | Low risk | Low risk |
| NCT00622284^11^ | Low risk | Low risk | Low risk | Low risk | Low risk | Low risk | Low risk |
| NCT00798161^12^ | Unknown | Unknown | Low risk | High risk | Low risk | Low risk | High risk |
| NCT01897532^13^ | Low risk | Low risk | Low risk | Low risk | Low risk | Low risk | Low risk |
| NCT01006603^14^ | Unknown | Low risk | Low risk | High risk | Low risk | Low risk | Low risk |
| NCT00757588^15,1615,16^ | Unknown | Low risk | Low risk | High risk | Low risk | Low risk | Low risk |
| NCT00575588^17,18^ | Unknown | Low risk | Low risk | High risk | Low risk | Low risk | High risk |
| NCT00121641^19,20^ | Unknown | Unknown | Low risk | High risk | Low risk | Low risk | High risk |
| NCT01107886^21^ | Unknown | Low risk | Low risk | Low risk | Low risk | Low risk | Low risk |
| NCT00121667^22^ | Unknown | Low risk | Low risk | High risk | Low risk | Low risk | Low risk |
| NCT00295633^23,24^ | Unknown | Low risk | Low risk | High risk | Low risk | Low risk | High risk |
| NCT00327015^25,26^ | Unknown | Low risk | Low risk | High risk | Low risk | Low risk | High risk |
| NCT00614939^27^ | Unknown | Low risk | Low risk | High risk | Low risk | Low risk | Low risk |
| NCT01289990^28,29^(1) | Low risk | Low risk | Low risk | High risk | Low risk | Low risk | Low risk |
| NCT00790205^30^ | Unknown | Low risk | Low risk | Low risk | Low risk | Low risk | High risk |
| NCT01098539^31^ | Unknown | Low risk | Low risk | Low risk | Low risk | Low risk | Low risk |
| NCT00509236^32^ | Low risk | Unknown | Low risk | Low risk | Low risk | Low risk | Low risk |
| NCT00509262^33^ | Low risk | Unknown | Low risk | Low risk | Low risk | Low risk | Low risk |
| NCT01046110^34^ | Unknown | Low risk | High risk | Low risk | Low risk | Low risk | Low risk |
| NCT00700817^35,36^ | Low risk | Low risk | High risk | High risk | Low risk | Low risk | Low risk |
| NCT00094770^37,38^ | Low risk | Unknown | Low risk | High risk | Low risk | Low risk | Low risk |
| NCT00395343^39^ | Low risk | Unknown | Low risk | High risk | Low risk | Low risk | High risk |
| NCT01189890^40^ | Low risk | Low risk | Low risk | High risk | Low risk | Low risk | High risk |
| NCT00838903^4141^ | Unknown | Unknown | Low risk | Low risk | Low risk | Low risk | Low risk |
| NCT00106340^42,43^ | Unknown | Unknown | Low risk | Low risk | Low risk | Low risk | High risk |
| NCT00849017^44^ | Unknown | Low risk | Low risk | Low risk | Low risk | Low risk | Low risk |
| NCT00839527^45^ | Unknown | Low risk | Low risk | Low risk | Low risk | Low risk | Low risk |
| NCT00849056^46^ | Unknown | Low risk | Low risk | Low risk | Low risk | Low risk | Low risk |
| NCT00838916^47^ | Low risk | Low risk | High risk | Low risk | Low risk | Low risk | Low risk |
| NCT01191268^48^ | Low risk | Low risk | High risk | Low risk | Low risk | Low risk | Low risk |
| NCT01126580^49^ | Unknown | Low risk | Low risk | High risk | Low risk | Low risk | Low risk |
| NCT01064687^50^ | Low risk | Low risk | High risk | High risk | Low risk | Low risk | Low risk |
| NCT00359762^51,52^ | Low risk | Unknown | High risk | High risk | Low risk | Low risk | Low risk |
| NCT00960661^53^ | Low risk | Unknown | High risk | High risk | Low risk | Low risk | Low risk |
| NCT00935532^54^ | Low risk | Low risk | High risk | High risk | Low risk | Low risk | Low risk |
| NCT01179048^55^ | Unknown | Low risk | Low risk | Low risk | Low risk | Low risk | Low risk |
| NCT00294723 | Unknown | Low risk | Low risk | High risk | Low risk | Low risk | Low risk |
| NCT00318461^59^ | Unknown | Low risk | Low risk | High risk | Low risk | Low risk | Low risk |
| NCT01147250^60^ | Unknown | Low risk | Low risk | Low risk | Low risk | Low risk | High risk |
| NCT00975286^61^ | Unknown | Low risk | Low risk | Low risk | Low risk | High risk | Low risk |
| NCT01106651^62,63^ | Low risk | Low risk | Low risk | High risk | Low risk | Low risk | Low risk |
| NCT00968812^64,65^ | Low risk | Low risk | Low risk | High risk | Low risk | Low risk | Low risk |
| NCT01064414^66,67^ | Unknown | Low risk | Low risk | High risk | Low risk | Low risk | Low risk |
| NCT01031680^68^ | Unknown | Unknown | Low risk | High risk | Low risk | Low risk | Low risk |
| NCT00660907^69,70,71^ | Low risk | Low risk | Low risk | High risk | Low risk | Low risk | High risk |
| NCT00663260^72^ | Unknown | Unknown | Low risk | High risk | Low risk | High risk | Low risk |
| NCT01042977^73^ | Unknown | Low risk | Low risk | High risk | Low risk | Low risk | Low risk |
| NCT00683878^74^ | Unknown | Unknown | Low risk | High risk | Low risk | Low risk | Low risk |
| NCT00680745^75^ | Low risk | Unknown | Low risk | High risk | Low risk | Low risk | High risk |
| NCT01730534^76^ | Unknown | Unknown | Low risk | Low risk | Low risk | Low risk | Low risk |
| NCT01131676^77^ | Low risk | Low risk | Low risk | Low risk | Low risk | Low risk | Low risk |
| NCT01368081^78^ | Low risk | Low risk | High risk | High risk | Low risk | Low risk | Low risk |
| NCT01164501^79^ | Low risk | Low risk | Low risk | High risk | Low risk | Low risk | Low risk |
| NCT01289990^28,29^(2) | Unknown | Low risk | Low risk | High risk | Low risk | Low risk | Low risk |
| NCT01167881^80^ | Low risk | Low risk | Low risk | High risk | Low risk | Low risk | Low risk |
| NCT01306214^81^ | Unknown | Low risk | Low risk | High risk | Low risk | Low risk | Low risk |
| NCT00521742^82^ | Unknown | Unknown | Low risk | Low risk | Low risk | High risk | High risk |
| 1998UKPDS^83^ | Low risk | Low risk | High risk | High risk | Low risk | Low risk | Low risk |
| 2008Hamann^84^ | Low risk | Low risk | Low risk | High risk | Low risk | High risk | Low risk |
| NCT00513630^85^ | Low risk | Low risk | Low risk | High risk | Low risk | Low risk | High risk |
| NCT00484198^86^ | Unknown | Unknown | Low risk | Low risk | Low risk | Low risk | High risk |
| NCT00116831^87^ | Unknown | Unknown | Low risk | Low risk | Low risk | Low risk | Low risk |
| NCT00494312^88^ | Unknown | Low risk | Low risk | High risk | Low risk | Low risk | Low risk |
| NCT00225277^89^ | Low risk | Low risk | Low risk | Low risk | Low risk | Low risk | Low risk |
| 2006Jain^90^ | Unknown | Unknown | Low risk | High risk | Low risk | High risk | Low risk |
| NCT00279045^91^ | Low risk | Low risk | Low risk | Low risk | Low risk | High risk | Low risk |
| 2005Charbonnel^92^ | Unknown | Low risk | Low risk | High risk | Low risk | High risk | High risk |
| NCT00099853^93^ | Unknown | Low risk | Low risk | Low risk | Low risk | Low risk | High risk |
| 2007Home^94^ | Unknown | Low risk | High risk | Low risk | Low risk | Low risk | Low risk |
| 2006Mazzone^95^ | Unknown | Low risk | Low risk | Low risk | Low risk | Low risk | High risk |
| NCT00770653^96^ | Unknown | Low risk | Low risk | High risk | Low risk | Low risk | High risk |
| 2008Chou^97^ | Unknown | Low risk | Low risk | High risk | Low risk | Low risk | High risk |
| 2005Matthews^98^ | Unknown | Low risk | Low risk | High risk | Low risk | Low risk | High risk |
| 2002St John Sutton^99^ | Unknown | High risk | High risk | High risk | Low risk | Low risk | Low risk |
| NCT01646320 | Unknown | High risk | High risk | Low risk | Low risk | Low risk | High risk |
| NCT01907854^101^ | Unknown | Low risk | Low risk | Low risk | Low risk | Low risk | Low risk |
| NCT02058147^102^ | Unknown | Low risk | High risk | Low risk | Low risk | Low risk | Low risk |
| NCT01768559^103^ | Unknown | Low risk | High risk | High risk | Low risk | Low risk | Low risk |
| NCT01032629 and NCT01989754^104^ | Low risk | Low risk | Low risk | Low risk | Low risk | Low risk | High risk |
| NCT01792518^105^ | Low risk | Low risk | Low risk | Low risk | Low risk | Low risk | Low risk |
| NCT01144338^106^ | Low risk | Low risk | Low risk | Low risk | Low risk | Low risk | Low risk |
| NCT02229383^107^ | Low risk | Low risk | Low risk | Low risk | Low risk | Low risk | Low risk |

| Table S5 Heart failure events of included studies | | | |
| --- | --- | --- | --- |
| Study | Arm | Total number of patients | Heart Failure |
| NCT00968708^1,2^ | Alogliptin | 2701 | 85 |
|  | Placebo | 2679 | 79 |
| NCT00856284^3^ | Alogliptin | 878 | 5 |
|  | Sulfonylurea | 869 | 2 |
| NCT00707993^4^ | Alogliptin | 222 | 3 |
|  | Sulfonylurea | 219 | 1 |
| NCT00328627^5^ | Alogliptin | 129 | 0 |
|  | Thiazolidinedione | 388 | 2 |
|  | Placebo | 129 | 0 |
| NCT00432276^6^ | Alogliptin | 404 | 2 |
|  | Thiazolidinedione | 399 | 1 |
| NCT00286494^8^ | Alogliptin | 199 | 2 |
|  | Placebo | 97 | 0 |
| NCT00800683^9^ | Linagliptin | 68 | 5 |
|  | Placebo | 65 | 1 |
| NCT00954447^10^ | Linagliptin | 631 | 4 |
|  | Placebo | 630 | 4 |
| NCT00622284^11^ | Linagliptin | 776 | 3 |
|  | Sulfonylurea | 775 | 2 |
| NCT00798161^12^ | Linagliptin | 142 | 1 |
|  | Metformin | 291 | 0 |
|  | Placebo | 72 | 0 |
| NCT01897532^13^ | Linagliptin | 3,494 | 209 |
|  | Placebo | 3,485 | 226 |
| NCT01006603^14^ | Saxagliptin | 360 | 1 |
|  | Sulfonylurea | 360 | 3 |
| NCT00757588^15,16^ | Saxagliptin | 304 | 2 |
|  | Placebo | 151 | 0 |
| NCT00575588^17,18^ | Saxagliptin | 428 | 1 |
|  | Sulfonylurea | 430 | 1 |
| NCT00121641^19,20^ | Saxagliptin | 208 | 1 |
|  | Placebo | 95 | 0 |
| NCT01107886^21^ | Saxagliptin | 8280 | 289 |
|  | Placebo | 8212 | 228 |
| NCT00121667^22^ | Saxagliptin | 383 | 3 |
|  | Placebo | 179 | 2 |
| NCT00295633^23,24^ | Saxagliptin | 381 | 0 |
|  | Placebo | 184 | 1 |
| NCT00327015^25,26^ | Saxagliptin | 320 | 0 |
|  | Placebo | 328 | 2 |
| NCT00614939^27^ | Saxagliptin | 85 | 1 |
|  | Placebo | 85 | 2 |
| NCT01289990^28,29^(1) | Placebo | 228 | 0 |
|  | Sitagliptin | 223 | 1 |
|  | Empagliflozin | 448 | 1 |
| NCT00790205^30^ | Sitagliptin | 7332 | 228 |
|  | Placebo | 7339 | 229 |
| NCT01098539^31^ | Sitagliptin | 246 | 1 |
|  | Albiglutide | 249 | 0 |
| NCT00509236^32^ | Sitagliptin | 64 | 2 |
|  | Sulfonylurea | 65 | 3 |
| NCT00509262^33^ | Sitagliptin | 210 | 0 |
|  | Sulfonylurea | 212 | 6 |
| NCT01046110^34^ | Sitagliptin | 226 | 0 |
|  | Insulin | 228 | 1 |
| NCT00700817^35,36^ | Sitagliptin | 219 | 0 |
|  | Liraglutide | 446 | 1 |
| NCT00094770^37,38^ | Sitagliptin | 588 | 2 |
|  | Sulfonylurea | 584 | 1 |
| NCT00395343^39^ | Sitagliptin | 322 | 0 |
|  | Placebo | 319 | 2 |
| NCT01189890^40^ | Sitagliptin | 241 | 0 |
|  | Sulfonylurea | 236 | 1 |
| NCT00838903^41^ | Sitagliptin | 302 | 1 |
|  | Placebo | 101 | 0 |
|  | Albiglutide | 302 | 3 |
|  | Sulfonylurea | 307 | 1 |
| NCT00106340^42,43^ | Vildagliptin | 1389/1533^*^ | 2 |
|  | Sulfonylurea | 1383/1546^*^ | 2 |
| NCT00849017^44^ | Albiglutide | 200 | 1 |
|  | Placebo | 101 | 3 |
| NCT00839527^45^ | Albiglutide | 271 | 0 |
|  | Thiazolidinedione | 277 | 4 |
|  | Placebo | 115 | 2 |
| NCT00849056^46^ | Albiglutide | 150 | 0 |
|  | Placebo | 151 | 1 |
| NCT00838916^47^ | Albiglutide | 504 | 2 |
|  | Insulin | 241 | 2 |
| NCT01191268^48^ | Dulaglutide | 588 | 2 |
|  | Insulin | 296 | 1 |
| NCT01126580^49^ | Dulaglutide | 539 | 1 |
|  | Metformin | 268 | 2 |
| NCT01064687^50^ | Dulaglutide | 559 | 1 |
|  | Exenatide | 278 | 0 |
|  | Placebo | 141 | 0 |
| NCT00359762^51,52^ | Exenatide | 511 | 1 |
|  | Sulfonylurea | 508 | 0 |
| NCT00960661^53^ | Exenatide | 315 | 1 |
|  | Insulin | 312 | 1 |
| NCT00935532^54^ | Exenatide | 215 | 1 |
|  | Insulin | 212 | 0 |
| NCT01179048^55^ | Placebo | 4672 | 248 |
|  | Liraglutide | 4668 | 218 |
| NCT00294723^56,57,58^ | Liraglutide | 497 | 1 |
|  | Sulfonylurea | 248 | 0 |
| NCT00318461^59^ | Liraglutide | 484 | 2 |
|  | Sulfonylurea | 242 | 0 |
|  | Placebo | 121 | 0 |
| NCT01147250^60^ | Lixisenatide | 3034 | 122 |
|  | Placebo | 3034 | 127 |
| NCT00975286^61^ | Lixisenatide | 223 | 0 |
|  | Placebo | 223 | 1 |
| NCT01106651^62,63^ | Canagliflozin | 477 | 1 |
|  | Placebo | 237 | 0 |
| NCT00968812^64,65^ | Canagliflozin | 968 | 0 |
|  | Sulfonylurea | 482 | 1 |
| NCT01064414^66,67^ | Canagliflozin | 179 | 3 |
|  | Placebo | 90 | 1 |
| NCT01031680^68^ | Dapagliflozin | 460 | 3 |
|  | Placebo | 462 | 5 |
| NCT00660907^69,70,71^ | Dapagliflozin | 406 | 1 |
|  | Sulfonylurea | 408 | 2 |
| NCT00663260^72^ | Dapagliflozin | 168 | 1 |
|  | Placebo | 84 | 1 |
| NCT01042977^73^ | Dapagliflozin | 482 | 1 |
|  | Placebo | 483 | 2 |
| NCT00683878^74^ | Dapagliflozin | 281 | 0 |
|  | Placebo | 139 | 1 |
| NCT00680745^75^ | Dapagliflozin | 296 | 0 |
|  | Placebo | 146 | 1 |
| NCT01730534^76^ | Dapagliflozin | 8,582 | 212 |
|  | Placebo | 8,578 | 286 |
| NCT01131676^77^ | Placebo | 2333 | 95 |
|  | Empagliflozin | 4687 | 125 |
| NCT01368081^78^ | Empagliflozin | 273 | 1 |
|  | Metformin | 63 | 0 |
| NCT01164501^79^ | Empagliflozin | 321 | 0 |
|  | Placebo | 319 | 4 |
| NCT01289990^28,29^（2） | Empagliflozin | 1202 | 4 |
|  | Placebo | 599 | 1 |
| NCT01167881^80^ | Empagliflozin | 765 | 2 |
|  | Sulfonylurea | 780 | 1 |
| NCT01306214^81^ | Empagliflozin | 375 | 2 |
|  | Placebo | 188 | 1 |
| NCT00521742^82^ | Glyburide | 152 | 7 |
|  | Pioglitazone | 149 | 10 |
| 1998UKPDS^83^ | Glibenclamide | 619 | 26 |
|  | Insulin | 911 | 25 |
| 2008Hamann^84^ | Glibenclamide or Gliclazide | 301 | 1 |
|  | Rosiglitazone | 294 | 1 |
| NCT00513630^85^ | Glipizide | 148 | 10 |
|  | Metformin | 156 | 9 |
| NCT00484198^86^ | Pioglitazone | 751 | 2 |
|  | Placebo | 137 | 0 |
| NCT00116831^87^ | Glipizide | 339 | 3 |
|  | Rosiglitazone | 333 | 8 |
| NCT00494312^88^ | Glibenclamide | 1057 | 11 |
|  | Pioglitazone | 1063 | 12 |
| NCT00225277^89^ | Glibenclamide | 273 | 5 |
|  | Pioglitazone | 270 | 4 |
| 2006Jain^90^ | Glyburide | 251 | 1 |
|  | Pioglitazone | 251 | 1 |
| NCT00279045^91^ | Glyburide | 1441 | 9 |
|  | Rosiglitazone | 1456 | 22 |
|  | Metformin | 1454 | 19 |
| 2005Charbonnel^92^ | Pioglitazone | 319 | 2 |
|  | Metformin | 320 | 3 |
| NCT00099853^93^ | Vildagliptin | 158 | 0 |
|  | Placebo | 158 | 1 |
| 2007Home^94^ | Sulfonylurea | 1105 | 18 |
|  | Rosiglitazone | 1117 | 25 |
|  | Rosiglitazone | 1103 | 23 |
|  | Metformin | 1122 | 13 |
| 2006Mazzone^95^ | Pioglitazone | 230 | 2 |
|  | Glimepiride | 228 | 0 |
| NCT00770653^96^ | Pioglitazone | 153 | 2 |
|  | Glimepiride | 149 | 0 |
| 2008Chou^97^ | Rosiglitazone | 230 | 2 |
|  | Glimepiride | 222 | 0 |
| 2005Matthews^98^ | Pioglitazone | 317 | 5 |
|  | Gliclazide | 313 | 2 |
| 2002St John Sutton^99^ | Rosiglitazone | 104 | 1 |
|  | Glyburide | 99 | 0 |
| NCT01646320^100^ | Dapagliflozin | 160 | 1 |
|  | Placebo | 160 | 0 |
| NCT01907854^101^ | Liraglutide | 202 | 1 |
|  | Sitagliptin | 204 | 0 |
| NCT02058147^102^ | Liraglutide | 233 | 0 |
|  | Insulin | 467 | 2 |
| NCT01768559^103^ | Lixisenatide | 298 | 0 |
|  | Insulin | 595 | 1 |
| NCT01032629 and NCT01989754^104^ | Canagliflozin | 5795 | 115 |
|  | Placebo | 4347 | 137 |
| NCT01792518^105^ | Linagliptin | 182 | 0 |
|  | Placebo | 178 | 2 |
| NCT01144338^106^ | Exenatide | 7356 | 219 |
|  | Placebo | 7396 | 231 |
| NCT02229383^107^ | Exenatide | 231 | 0 |
|  | Placebo | 229 | 2 |

**References:**

1. Zannad F, Cannon CP, Cushman WC, Bakris GL, Menon V, Perez AT, Fleck PR, Mehta CR, Kupfer S, Wilson C, Lam H, White WB. Heart failure and mortality outcomes in patients with type 2 diabetes taking alogliptin versus placebo in EXAMINE: a multicentre, randomised, double-blind trial. *Lancet* 2015; **385**:2067-2076.

2. White WB, Cannon CP, Heller SR, Nissen SE, Bergenstal RM, Bakris GL, Perez AT, Fleck PR, Mehta CR, Kupfer S, Wilson C, Cushman WC, Zannad F. Alogliptin after acute coronary syndrome in patients with type 2 diabetes. *N Engl J Med* 2013; **369**:1327-1335.

3. Del PS, Camisasca R, Wilson C, Fleck P. Durability of the efficacy and safety of alogliptin compared with glipizide in type 2 diabetes mellitus: a 2-year study. *Diabetes Obes Metab* 2014; **16**:1239-1246.

4. Rosenstock J, Wilson C, Fleck P. Alogliptin versus glipizide monotherapy in elderly type 2 diabetes mellitus patients with mild hyperglycaemia: a prospective, double-blind, randomized, 1-year study. *Diabetes Obes Metab* 2013; **15**:906-914.

5. DeFronzo RA, Burant CF, Fleck P, Wilson C, Mekki Q, Pratley RE. Efficacy and tolerability of the DPP-4 inhibitor alogliptin combined with pioglitazone, in metformin-treated patients with type 2 diabetes. *J Clin Endocrinol Metab* 2012; **97**:1615-1622.

6. Bosi E, Ellis GC, Wilson CA, Fleck PR. Alogliptin as a third oral antidiabetic drug in patients with type 2 diabetes and inadequate glycaemic control on metformin and pioglitazone: a 52-week, randomized, double-blind, active-controlled, parallel-group study. *Diabetes Obes Metab* 2011; **13**:1088-1096.

7. Nauck MA, Ellis GC, Fleck PR, Wilson CA, Mekki Q. Efficacy and safety of adding the dipeptidyl peptidase-4 inhibitor alogliptin to metformin therapy in patients with type 2 diabetes inadequately controlled with metformin monotherapy: a multicentre, randomised, double-blind, placebo-controlled study. *Int J Clin Pract* 2009; **63**:46-55.

8. Pratley RE, Reusch JE, Fleck PR, Wilson CA, Mekki Q. Efficacy and safety of the dipeptidyl peptidase-4 inhibitor alogliptin added to pioglitazone in patients with type 2 diabetes: a randomized, double-blind, placebo-controlled study. *Curr Med Res Opin* 2009; **25**:2361-2371.

9. McGill JB, Sloan L, Newman J, Patel S, Sauce C, von Eynatten M, Woerle HJ. Long-term efficacy and safety of linagliptin in patients with type 2 diabetes and severe renal impairment: a 1-year, randomized, double-blind, placebo-controlled study. *Diabetes Care* 2013; **36**:237-244.

10. Yki-Jarvinen H, Rosenstock J, Duran-Garcia S, Pinnetti S, Bhattacharya S, Thiemann S, Patel S, Woerle HJ. Effects of adding linagliptin to basal insulin regimen for inadequately controlled type 2 diabetes: a >/=52-week randomized, double-blind study. *Diabetes Care* 2013; **36**:3875-3881.

11. Gallwitz B, Rosenstock J, Rauch T, Bhattacharya S, Patel S, von Eynatten M, Dugi KA, Woerle HJ. 2-year efficacy and safety of linagliptin compared with glimepiride in patients with type 2 diabetes inadequately controlled on metformin: a randomised, double-blind, non-inferiority trial. *Lancet* 2012; **380**:475-483.

12. Haak T, Meinicke T, Jones R, Weber S, von Eynatten M, Woerle HJ. Initial combination of linagliptin and metformin improves glycaemic control in type 2 diabetes: a randomized, double-blind, placebo-controlled study. *Diabetes Obes Metab* 2012; **14**:565-574.

13. Rosenstock J, Perkovic V, Johansen OE, Cooper ME, Kahn SE, Marx N, Alexander JH, Pencina M, Toto RD, Wanner C, Zinman B, Woerle HJ, Baanstra D, Pfarr E, Schnaidt S, Meinicke T, George JT, von Eynatten M, McGuire DK. Effect of Linagliptin vs Placebo on Major Cardiovascular Events in Adults With Type 2 Diabetes and High Cardiovascular and Renal Risk: The CARMELINA Randomized Clinical Trial. *JAMA* 2019; **321**:69-79.

14. Schernthaner G, Duran-Garcia S, Hanefeld M, Langslet G, Niskanen L, Ostgren CJ, Malvolti E, Hardy E. Efficacy and tolerability of saxagliptin compared with glimepiride in elderly patients with type 2 diabetes: a randomized, controlled study (GENERATION). *Diabetes Obes Metab* 2015; **17**:630-638.

15. Barnett AH, Charbonnel B, Li J, Donovan M, Fleming D, Iqbal N. Saxagliptin add-on therapy to insulin with or without metformin for type 2 diabetes mellitus: 52-week safety and efficacy. *Clin Drug Investig* 2013; **33**:707-717.

16. Barnett AH, Charbonnel B, Donovan M, Fleming D, Chen R. Effect of saxagliptin as add-on therapy in patients with poorly controlled type 2 diabetes on insulin alone or insulin combined with metformin. *Curr Med Res Opin* 2012; **28**:513-523.

17. Goke B, Gallwitz B, Eriksson JG, Hellqvist A, Gause-Nilsson I. Saxagliptin vs. glipizide as add-on therapy in patients with type 2 diabetes mellitus inadequately controlled on metformin alone: long-term (52-week) extension of a 52-week randomised controlled trial. *Int J Clin Pract* 2013; **67**:307-316.

18. Goke B, Gallwitz B, Eriksson J, Hellqvist A, Gause-Nilsson I. Saxagliptin is non-inferior to glipizide in patients with type 2 diabetes mellitus inadequately controlled on metformin alone: a 52-week randomised controlled trial. *Int J Clin Pract* 2010; **64**:1619-1631.

19. Rosenstock J, Gross JL, Aguilar-Salinas C, Hissa M, Berglind N, Ravichandran S, Fleming D. Long-term 4-year safety of saxagliptin in drug-naive and metformin-treated patients with Type 2 diabetes. *Diabet Med* 2013; **30**:1472-1476.

20. Rosenstock J, Aguilar-Salinas C, Klein E, Nepal S, List J, Chen R. Effect of saxagliptin monotherapy in treatment-naive patients with type 2 diabetes. *Curr Med Res Opin* 2009; **25**:2401-2411.

21. Scirica BM, Bhatt DL, Braunwald E, Steg PG, Davidson J, Hirshberg B, Ohman P, Frederich R, Wiviott SD, Hoffman EB, Cavender MA, Udell JA, Desai NR, Mosenzon O, McGuire DK, Ray KK, Leiter LA, Raz I. Saxagliptin and cardiovascular outcomes in patients with type 2 diabetes mellitus. *N Engl J Med* 2013; **369**:1317-1326.

22. DeFronzo RA, Hissa MN, Garber AJ, Luiz GJ, Yuyan DR, Ravichandran S, Chen RS. The efficacy and safety of saxagliptin when added to metformin therapy in patients with inadequately controlled type 2 diabetes with metformin alone. *Diabetes Care* 2009; **32**:1649-1655.

23. Hollander PL, Li J, Frederich R, Allen E, Chen R. Safety and efficacy of saxagliptin added to thiazolidinedione over 76 weeks in patients with type 2 diabetes mellitus. *Diab Vasc Dis Res* 2011; **8**:125-135.

24. Hollander P, Li J, Allen E, Chen R. Saxagliptin added to a thiazolidinedione improves glycemic control in patients with type 2 diabetes and inadequate control on thiazolidinedione alone. *J Clin Endocrinol Metab* 2009; **94**:4810-4819.

25. Pfutzner A, Paz-Pacheco E, Allen E, Frederich R, Chen R. Initial combination therapy with saxagliptin and metformin provides sustained glycaemic control and is well tolerated for up to 76 weeks. *Diabetes Obes Metab* 2011; **13**:567-576.

26. Jadzinsky M, Pfutzner A, Paz-Pacheco E, Xu Z, Allen E, Chen R. Saxagliptin given in combination with metformin as initial therapy improves glycaemic control in patients with type 2 diabetes compared with either monotherapy: a randomized controlled trial. *Diabetes Obes Metab* 2009; **11**:611-622.

27. Nowicki M, Rychlik I, Haller H, Warren M, Suchower L, Gause-Nilsson I, Schutzer KM. Long-term treatment with the dipeptidyl peptidase-4 inhibitor saxagliptin in patients with type 2 diabetes mellitus and renal impairment: a randomised controlled 52-week efficacy and safety study. *Int J Clin Pract* 2011; **65**:1230-1239.

28. Haering HU, Merker L, Christiansen AV, Roux F, Salsali A, Kim G, Meinicke T, Woerle HJ, Broedl UC. Empagliflozin as add-on to metformin plus sulphonylurea in patients with type 2 diabetes. *Diabetes Res Clin Pract* 2015; **110**:82-90.

29. Roden M, Merker L, Christiansen AV, Roux F, Salsali A, Kim G, Stella P, Woerle HJ, Broedl UC. Safety, tolerability and effects on cardiometabolic risk factors of empagliflozin monotherapy in drug-naive patients with type 2 diabetes: a double-blind extension of a Phase III randomized controlled trial. *Cardiovasc Diabetol* 2015; **14**:154.

30. Green JB, Bethel MA, Armstrong PW, Buse JB, Engel SS, Garg J, Josse R, Kaufman KD, Koglin J, Korn S, Lachin JM, McGuire DK, Pencina MJ, Standl E, Stein PP, Suryawanshi S, Van de Werf F, Peterson ED, Holman RR. Effect of Sitagliptin on Cardiovascular Outcomes in Type 2 Diabetes. *N Engl J Med* 2015; **373**:232-242.

31. Leiter LA, Carr MC, Stewart M, Jones-Leone A, Scott R, Yang F, Handelsman Y. Efficacy and safety of the once-weekly GLP-1 receptor agonist albiglutide versus sitagliptin in patients with type 2 diabetes and renal impairment: a randomized phase III study. *Diabetes Care* 2014; **37**:2723-2730.

32. Arjona FJ, Corry D, Mogensen CE, Sloan L, Xu L, Golm GT, Gonzalez EJ, Davies MJ, Kaufman KD, Goldstein BJ. Efficacy and safety of sitagliptin in patients with type 2 diabetes and ESRD receiving dialysis: a 54-week randomized trial. *Am J Kidney Dis* 2013; **61**:579-587.

33. Arjona FJ, Marre M, Barzilai N, Guo H, Golm GT, Sisk CM, Kaufman KD, Goldstein BJ. Efficacy and safety of sitagliptin versus glipizide in patients with type 2 diabetes and moderate-to-severe chronic renal insufficiency. *Diabetes Care* 2013; **36**:1067-1073.

34. Philis-Tsimikas A, Del PS, Satman I, Bhargava A, Dharmalingam M, Skjoth TV, Rasmussen S, Garber AJ. Effect of insulin degludec versus sitagliptin in patients with type 2 diabetes uncontrolled on oral antidiabetic agents. *Diabetes Obes Metab* 2013; **15**:760-766.

35. Pratley R, Nauck M, Bailey T, Montanya E, Cuddihy R, Filetti S, Garber A, Thomsen AB, Hartvig H, Davies M. One year of liraglutide treatment offers sustained and more effective glycaemic control and weight reduction compared with sitagliptin, both in combination with metformin, in patients with type 2 diabetes: a randomised, parallel-group, open-label trial. *Int J Clin Pract* 2011; **65**:397-407.

36. Pratley RE, Nauck M, Bailey T, Montanya E, Cuddihy R, Filetti S, Thomsen AB, Sondergaard RE, Davies M. Liraglutide versus sitagliptin for patients with type 2 diabetes who did not have adequate glycaemic control with metformin: a 26-week, randomised, parallel-group, open-label trial. *Lancet* 2010; **375**:1447-1456.

37. Seck T, Nauck M, Sheng D, Sunga S, Davies MJ, Stein PP, Kaufman KD, Amatruda JM. Safety and efficacy of treatment with sitagliptin or glipizide in patients with type 2 diabetes inadequately controlled on metformin: a 2-year study. *Int J Clin Pract* 2010; **64**:562-576.

38. Nauck MA, Meininger G, Sheng D, Terranella L, Stein PP. Efficacy and safety of the dipeptidyl peptidase-4 inhibitor, sitagliptin, compared with the sulfonylurea, glipizide, in patients with type 2 diabetes inadequately controlled on metformin alone: a randomized, double-blind, non-inferiority trial. *Diabetes Obes Metab* 2007; **9**:194-205.

39. Vilsboll T, Rosenstock J, Yki-Jarvinen H, Cefalu WT, Chen Y, Luo E, Musser B, Andryuk PJ, Ling Y, Kaufman KD, Amatruda JM, Engel SS, Katz L. Efficacy and safety of sitagliptin when added to insulin therapy in patients with type 2 diabetes. *Diabetes Obes Metab* 2010; **12**:167-177.

40. Hartley P, Shentu Y, Betz-Schiff P, Golm GT, Sisk CM, Engel SS, Shankar RR. Efficacy and Tolerability of Sitagliptin Compared with Glimepiride in Elderly Patients with Type 2 Diabetes Mellitus and Inadequate Glycemic Control: A Randomized, Double-Blind, Non-Inferiority Trial. *Drugs Aging* 2015; **32**:469-476.

41. Ahren B, Johnson SL, Stewart M, Cirkel DT, Yang F, Perry C, Feinglos MN. HARMONY 3: 104-week randomized, double-blind, placebo- and active-controlled trial assessing the efficacy and safety of albiglutide compared with placebo, sitagliptin, and glimepiride in patients with type 2 diabetes taking metformin. *Diabetes Care* 2014; **37**:2141-2148.

42. Ferrannini E, Fonseca V, Zinman B, Matthews D, Ahren B, Byiers S, Shao Q, Dejager S. Fifty-two-week efficacy and safety of vildagliptin vs. glimepiride in patients with type 2 diabetes mellitus inadequately controlled on metformin monotherapy. *Diabetes Obes Metab* 2009; **11**:157-166.

43. Matthews DR, Dejager S, Ahren B, Fonseca V, Ferrannini E, Couturier A, Foley JE, Zinman B. Vildagliptin add-on to metformin produces similar efficacy and reduced hypoglycaemic risk compared with glimepiride, with no weight gain: results from a 2-year study. *Diabetes Obes Metab* 2010; **12**:780-789.

44. Nauck MA, Stewart MW, Perkins C, Jones-Leone A, Yang F, Perry C, Reinhardt RR, Rendell M. Efficacy and safety of once-weekly GLP-1 receptor agonist albiglutide (HARMONY 2): 52 week primary endpoint results from a randomised, placebo-controlled trial in patients with type 2 diabetes mellitus inadequately controlled with diet and exercise. *Diabetologia* 2016; **59**:266-274.

45. Home PD, Shamanna P, Stewart M, Yang F, Miller M, Perry C, Carr MC. Efficacy and tolerability of albiglutide versus placebo or pioglitazone over 1 year in people with type 2 diabetes currently taking metformin and glimepiride: HARMONY 5. *Diabetes Obes Metab* 2015; **17**:179-187.

46. Reusch J, Stewart MW, Perkins CM, Cirkel DT, Ye J, Perry CR, Reinhardt RR, Bode BW. Efficacy and safety of once-weekly glucagon-like peptide 1 receptor agonist albiglutide (HARMONY 1 trial): 52-week primary endpoint results from a randomized, double-blind, placebo-controlled trial in patients with type 2 diabetes mellitus not controlled on pioglitazone, with or without metformin. *Diabetes Obes Metab* 2014; **16**:1257-1264.

47. Weissman PN, Carr MC, Ye J, Cirkel DT, Stewart M, Perry C, Pratley R. HARMONY 4: randomised clinical trial comparing once-weekly albiglutide and insulin glargine in patients with type 2 diabetes inadequately controlled with metformin with or without sulfonylurea. *Diabetologia* 2014; **57**:2475-2484.

48. Blonde L, Jendle J, Gross J, Woo V, Jiang H, Fahrbach JL, Milicevic Z. Once-weekly dulaglutide versus bedtime insulin glargine, both in combination with prandial insulin lispro, in patients with type 2 diabetes (AWARD-4): a randomised, open-label, phase 3, non-inferiority study. *Lancet* 2015; **385**:2057-2066.

49. Umpierrez G, Tofe PS, Perez MF, Shurzinske L, Pechtner V. Efficacy and safety of dulaglutide monotherapy versus metformin in type 2 diabetes in a randomized controlled trial (AWARD-3). *Diabetes Care* 2014; **37**:2168-2176.

50. Wysham C, Blevins T, Arakaki R, Colon G, Garcia P, Atisso C, Kuhstoss D, Lakshmanan M. Efficacy and safety of dulaglutide added onto pioglitazone and metformin versus exenatide in type 2 diabetes in a randomized controlled trial (AWARD-1). *Diabetes Care* 2014; **37**:2159-2167.

51. Simo R, Guerci B, Schernthaner G, Gallwitz B, Rosas-Guzman J, Dotta F, Festa A, Zhou M, Kiljanski J. Long-term changes in cardiovascular risk markers during administration of exenatide twice daily or glimepiride: results from the European exenatide study. *Cardiovasc Diabetol* 2015; **14**:116.

52. Gallwitz B, Guzman J, Dotta F, Guerci B, Simo R, Basson BR, Festa A, Kiljanski J, Sapin H, Trautmann M, Schernthaner G. Exenatide twice daily versus glimepiride for prevention of glycaemic deterioration in patients with type 2 diabetes with metformin failure (EUREXA): an open-label, randomised controlled trial. *Lancet* 2012; **379**:2270-2278.

53. Diamant M, Nauck MA, Shaginian R, Malone JK, Cleall S, Reaney M, de Vries D, Hoogwerf BJ, MacConell L, Wolffenbuttel BH. Glucagon-like peptide 1 receptor agonist or bolus insulin with optimized basal insulin in type 2 diabetes. *Diabetes Care* 2014; **37**:2763-2773.

54. Inagaki N, Atsumi Y, Oura T, Saito H, Imaoka T. Efficacy and safety profile of exenatide once weekly compared with insulin once daily in Japanese patients with type 2 diabetes treated with oral antidiabetes drug(s): results from a 26-week, randomized, open-label, parallel-group, multicenter, noninferiority study. *Clin Ther* 2012; **34**:1892-1908.

55. Marso SP, Daniels GH, Brown-Frandsen K, Kristensen P, Mann JF, Nauck MA, Nissen SE, Pocock S, Poulter NR, Ravn LS, Steinberg WM, Stockner M, Zinman B, Bergenstal RM, Buse JB. Liraglutide and Cardiovascular Outcomes in Type 2 Diabetes. *N Engl J Med* 2016.

56. Garber A, Henry RR, Ratner R, Hale P, Chang CT, Bode B. Liraglutide, a once-daily human glucagon-like peptide 1 analogue, provides sustained improvements in glycaemic control and weight for 2 years as monotherapy compared with glimepiride in patients with type 2 diabetes. *Diabetes Obes Metab* 2011; **13**:348-356.

57. Bode BW, Testa MA, Magwire M, Hale PM, Hammer M, Blonde L, Garber A. Patient-reported outcomes following treatment with the human GLP-1 analogue liraglutide or glimepiride in monotherapy: results from a randomized controlled trial in patients with type 2 diabetes. *Diabetes Obes Metab* 2010; **12**:604-612.

58. Garber A, Henry R, Ratner R, Garcia-Hernandez PA, Rodriguez-Pattzi H, Olvera-Alvarez I, Hale PM, Zdravkovic M, Bode B. Liraglutide versus glimepiride monotherapy for type 2 diabetes (LEAD-3 Mono): a randomised, 52-week, phase III, double-blind, parallel-treatment trial. *Lancet* 2009; **373**:473-481.

59. Nauck M, Frid A, Hermansen K, Shah NS, Tankova T, Mitha IH, Zdravkovic M, During M, Matthews DR. Efficacy and safety comparison of liraglutide, glimepiride, and placebo, all in combination with metformin, in type 2 diabetes: the LEAD (liraglutide effect and action in diabetes)-2 study. *Diabetes Care* 2009; **32**:84-90.

60. Pfeffer MA, Claggett B, Diaz R, Dickstein K, Gerstein HC, Kober LV, Lawson FC, Ping L, Wei X, Lewis EF, Maggioni AP, McMurray JJ, Probstfield JL, Riddle MC, Solomon SD, Tardif JC. Lixisenatide in Patients with Type 2 Diabetes and Acute Coronary Syndrome. *N Engl J Med* 2015; **373**:2247-2257.

61. Riddle MC, Forst T, Aronson R, Sauque-Reyna L, Souhami E, Silvestre L, Ping L, Rosenstock J. Adding once-daily lixisenatide for type 2 diabetes inadequately controlled with newly initiated and continuously titrated basal insulin glargine: a 24-week, randomized, placebo-controlled study (GetGoal-Duo 1). *Diabetes Care* 2013; **36**:2497-2503.

62. Bode B, Stenlof K, Harris S, Sullivan D, Fung A, Usiskin K, Meininger G. Long-term efficacy and safety of canagliflozin over 104 weeks in patients aged 55-80 years with type 2 diabetes. *Diabetes Obes Metab* 2015; **17**:294-303.

63. Bode B, Stenlof K, Sullivan D, Fung A, Usiskin K. Efficacy and safety of canagliflozin treatment in older subjects with type 2 diabetes mellitus: a randomized trial. *Hosp Pract (1995)* 2013; **41**:72-84.

64. Leiter LA, Yoon KH, Arias P, Langslet G, Xie J, Balis DA, Millington D, Vercruysse F, Canovatchel W, Meininger G. Canagliflozin provides durable glycemic improvements and body weight reduction over 104 weeks versus glimepiride in patients with type 2 diabetes on metformin: a randomized, double-blind, phase 3 study. *Diabetes Care* 2015; **38**:355-364.

65. Cefalu WT, Leiter LA, Yoon KH, Arias P, Niskanen L, Xie J, Balis DA, Canovatchel W, Meininger G. Efficacy and safety of canagliflozin versus glimepiride in patients with type 2 diabetes inadequately controlled with metformin (CANTATA-SU): 52 week results from a randomised, double-blind, phase 3 non-inferiority trial. *Lancet* 2013; **382**:941-950.

66. Yale JF, Bakris G, Cariou B, Nieto J, David-Neto E, Yue D, Wajs E, Figueroa K, Jiang J, Law G, Usiskin K, Meininger G. Efficacy and safety of canagliflozin over 52 weeks in patients with type 2 diabetes mellitus and chronic kidney disease. *Diabetes Obes Metab* 2014; **16**:1016-1027.

67. Yale JF, Bakris G, Cariou B, Yue D, David-Neto E, Xi L, Figueroa K, Wajs E, Usiskin K, Meininger G. Efficacy and safety of canagliflozin in subjects with type 2 diabetes and chronic kidney disease. *Diabetes Obes Metab* 2013; **15**:463-473.

68. Cefalu WT, Leiter LA, de Bruin TW, Gause-Nilsson I, Sugg J, Parikh SJ. Dapagliflozin's Effects on Glycemia and Cardiovascular Risk Factors in High-Risk Patients With Type 2 Diabetes: A 24-Week, Multicenter, Randomized, Double-Blind, Placebo-Controlled Study With a 28-Week Extension. *Diabetes Care* 2015; **38**:1218-1227.

69. Del PS, Nauck M, Duran-Garcia S, Maffei L, Rohwedder K, Theuerkauf A, Parikh S. Long-term glycaemic response and tolerability of dapagliflozin versus a sulphonylurea as add-on therapy to metformin in patients with type 2 diabetes: 4-year data. *Diabetes Obes Metab* 2015; **17**:581-590.

70. Nauck MA, Del PS, Duran-Garcia S, Rohwedder K, Langkilde AM, Sugg J, Parikh SJ. Durability of glycaemic efficacy over 2 years with dapagliflozin versus glipizide as add-on therapies in patients whose type 2 diabetes mellitus is inadequately controlled with metformin. *Diabetes Obes Metab* 2014; **16**:1111-1120.

71. Nauck MA, Del PS, Meier JJ, Duran-Garcia S, Rohwedder K, Elze M, Parikh SJ. Dapagliflozin versus glipizide as add-on therapy in patients with type 2 diabetes who have inadequate glycemic control with metformin: a randomized, 52-week, double-blind, active-controlled noninferiority trial. *Diabetes Care* 2011; **34**:2015-2022.

72. Kohan DE, Fioretto P, Tang W, List JF. Long-term study of patients with type 2 diabetes and moderate renal impairment shows that dapagliflozin reduces weight and blood pressure but does not improve glycemic control. *Kidney Int* 2014; **85**:962-971.

73. Leiter LA, Cefalu WT, de Bruin TW, Gause-Nilsson I, Sugg J, Parikh SJ. Dapagliflozin added to usual care in individuals with type 2 diabetes mellitus with preexisting cardiovascular disease: a 24-week, multicenter, randomized, double-blind, placebo-controlled study with a 28-week extension. *J Am Geriatr Soc* 2014; **62**:1252-1262.

74. Rosenstock J, Vico M, Wei L, Salsali A, List JF. Effects of dapagliflozin, an SGLT2 inhibitor, on HbA(1c), body weight, and hypoglycemia risk in patients with type 2 diabetes inadequately controlled on pioglitazone monotherapy. *Diabetes Care* 2012; **35**:1473-1478.

75. Strojek K, Yoon KH, Hruba V, Elze M, Langkilde AM, Parikh S. Effect of dapagliflozin in patients with type 2 diabetes who have inadequate glycaemic control with glimepiride: a randomized, 24-week, double-blind, placebo-controlled trial. *Diabetes Obes Metab* 2011; **13**:928-938.

76. Wiviott SD, Raz I, Bonaca MP, Mosenzon O, Kato ET, Cahn A, Silverman MG, Zelniker TA, Kuder JF, Murphy SA, Bhatt DL, Leiter LA, McGuire DK, Wilding J, Ruff CT, Gause-Nilsson I, Fredriksson M, Johansson PA, Langkilde AM, Sabatine MS. Dapagliflozin and Cardiovascular Outcomes in Type 2 Diabetes. *N Engl J Med* 2019; **380**:347-357.

77. Zinman B, Wanner C, Lachin JM, Fitchett D, Bluhmki E, Hantel S, Mattheus M, Devins T, Johansen OE, Woerle HJ, Broedl UC, Inzucchi SE. Empagliflozin, Cardiovascular Outcomes, and Mortality in Type 2 Diabetes. *N Engl J Med* 2015; **373**:2117-2128.

78. Araki E, Tanizawa Y, Tanaka Y, Taniguchi A, Koiwai K, Kim G, Salsali A, Woerle HJ, Broedl UC. Long-term treatment with empagliflozin as add-on to oral antidiabetes therapy in Japanese patients with type 2 diabetes mellitus. *Diabetes Obes Metab* 2015; **17**:665-674.

79. Barnett AH, Mithal A, Manassie J, Jones R, Rattunde H, Woerle HJ, Broedl UC. Efficacy and safety of empagliflozin added to existing antidiabetes treatment in patients with type 2 diabetes and chronic kidney disease: a randomised, double-blind, placebo-controlled trial. *Lancet Diabetes Endocrinol* 2014; **2**:369-384.

80. Ridderstrale M, Andersen KR, Zeller C, Kim G, Woerle HJ, Broedl UC. Comparison of empagliflozin and glimepiride as add-on to metformin in patients with type 2 diabetes: a 104-week randomised, active-controlled, double-blind, phase 3 trial. *Lancet Diabetes Endocrinol* 2014; **2**:691-700.

81. Rosenstock J, Jelaska A, Frappin G, Salsali A, Kim G, Woerle HJ, Broedl UC. Improved glucose control with weight loss, lower insulin doses, and no increased hypoglycemia with empagliflozin added to titrated multiple daily injections of insulin in obese inadequately controlled type 2 diabetes. *Diabetes Care* 2014; **37**:1815-1823.

82. Giles TD, Elkayam U, Bhattacharya M, Perez A, Miller AB. Comparison of pioglitazone vs glyburide in early heart failure: insights from a randomized controlled study of patients with type 2 diabetes and mild cardiac disease. *Congest Heart Fail* 2010; **16**:111-117.

83. Intensive blood-glucose control with sulphonylureas or insulin compared with conventional treatment and risk of complications in patients with type 2 diabetes (UKPDS 33). UK Prospective Diabetes Study (UKPDS) Group. *Lancet* 1998; **352**:837-853.

84. Hamann A, Garcia-Puig J, Paul G, Donaldson J, Stewart M. Comparison of fixed-dose rosiglitazone/metformin combination therapy with sulphonylurea plus metformin in overweight individuals with Type 2 diabetes inadequately controlled on metformin alone. *Exp Clin Endocrinol Diabetes* 2008; **116**:6-13.

85. Hong J, Zhang Y, Lai S, Lv A, Su Q, Dong Y, Zhou Z, Tang W, Zhao J, Cui L, Zou D, Wang D, Li H, Liu C, Wu G, Shen J, Zhu D, Wang W, Shen W, Ning G. Effects of metformin versus glipizide on cardiovascular outcomes in patients with type 2 diabetes and coronary artery disease. *Diabetes Care* 2013; **36**:1304-1311.

86. Chou HS, Truitt KE, Moberly JB, Merante D, Choi Y, Mun Y, Pfutzner A. A 26-week, placebo- and pioglitazone-controlled monotherapy study of rivoglitazone in subjects with type 2 diabetes mellitus. *Diabetes Obes Metab* 2012; **14**:1000-1009.

87. Gerstein HC, Ratner RE, Cannon CP, Serruys PW, Garcia-Garcia HM, van Es GA, Kolatkar NS, Kravitz BG, Miller DM, Huang C, Fitzgerald PJ, Nesto RW. Effect of rosiglitazone on progression of coronary atherosclerosis in patients with type 2 diabetes mellitus and coronary artery disease: the assessment on the prevention of progression by rosiglitazone on atherosclerosis in diabetes patients with cardiovascular history trial. *Circulation* 2010; **121**:1176-1187.

88. Tolman KG, Freston JW, Kupfer S, Perez A. Liver safety in patients with type 2 diabetes treated with pioglitazone: results from a 3-year, randomized, comparator-controlled study in the US. *Drug Saf* 2009; **32**:787-800.

89. Nissen SE, Nicholls SJ, Wolski K, Nesto R, Kupfer S, Perez A, Jure H, De Larochelliere R, Staniloae CS, Mavromatis K, Saw J, Hu B, Lincoff AM, Tuzcu EM. Comparison of pioglitazone vs glimepiride on progression of coronary atherosclerosis in patients with type 2 diabetes: the PERISCOPE randomized controlled trial. *JAMA* 2008; **299**:1561-1573.

90. Jain R, Osei K, Kupfer S, Perez AT, Zhang J. Long-term safety of pioglitazone versus glyburide in patients with recently diagnosed type 2 diabetes mellitus. *Pharmacotherapy* 2006; **26**:1388-1395.

91. Kahn SE, Haffner SM, Heise MA, Herman WH, Holman RR, Jones NP, Kravitz BG, Lachin JM, O'Neill MC, Zinman B, Viberti G. Glycemic durability of rosiglitazone, metformin, or glyburide monotherapy. *N Engl J Med* 2006; **355**:2427-2443.

92. Charbonnel B, Schernthaner G, Brunetti P, Matthews DR, Urquhart R, Tan MH, Hanefeld M. Long-term efficacy and tolerability of add-on pioglitazone therapy to failing monotherapy compared with addition of gliclazide or metformin in patients with type 2 diabetes. *Diabetologia* 2005; **48**:1093-1104.

93. Garber AJ, Schweizer A, Baron MA, Rochotte E, Dejager S. Vildagliptin in combination with pioglitazone improves glycaemic control in patients with type 2 diabetes failing thiazolidinedione monotherapy: a randomized, placebo-controlled study. *Diabetes Obes Metab* 2007; **9**:166-174.

94. Home PD, Pocock SJ, Beck-Nielsen H, Gomis R, Hanefeld M, Jones NP, Komajda M, McMurray JJ. Rosiglitazone evaluated for cardiovascular outcomes--an interim analysis. *N Engl J Med* 2007; **357**:28-38.

95. Mazzone T, Meyer PM, Feinstein SB, Davidson MH, Kondos GT, D'Agostino RS, Perez A, Provost JC, Haffner SM. Effect of pioglitazone compared with glimepiride on carotid intima-media thickness in type 2 diabetes: a randomized trial. *JAMA* 2006; **296**:2572-2581.

96. Pfutzner A, Schondorf T, Tschope D, Lobmann R, Merke J, Muller J, Lehmann U, Fuchs W, Forst T. PIOfix-study: effects of pioglitazone/metformin fixed combination in comparison with a combination of metformin with glimepiride on diabetic dyslipidemia. *Diabetes Technol Ther* 2011; **13**:637-643.

97. Chou HS, Palmer JP, Jones AR, Waterhouse B, Ferreira-Cornwell C, Krebs J, Goldstein BJ. Initial treatment with fixed-dose combination rosiglitazone/glimepiride in patients with previously untreated type 2 diabetes. *Diabetes Obes Metab* 2008; **10**:626-637.

98. Matthews DR, Charbonnel BH, Hanefeld M, Brunetti P, Schernthaner G. Long-term therapy with addition of pioglitazone to metformin compared with the addition of gliclazide to metformin in patients with type 2 diabetes: a randomized, comparative study. *Diabetes Metab Res Rev* 2005; **21**:167-174.

99. St JSM, Rendell M, Dandona P, Dole JF, Murphy K, Patwardhan R, Patel J, Freed M. A comparison of the effects of rosiglitazone and glyburide on cardiovascular function and glycemic control in patients with type 2 diabetes. *Diabetes Care* 2002; **25**:2058-2064.

100. Mathieu C, Ranetti AE, Li D, Ekholm E, Cook W, Hirshberg B, Chen H, Hansen L, Iqbal N. Randomized, Double-Blind, Phase 3 Trial of Triple Therapy With Dapagliflozin Add-on to Saxagliptin Plus Metformin in Type 2 Diabetes. *Diabetes Care* 2015; **38**:2009-2017.

101. Bailey TS, Takacs R, Tinahones FJ, Rao PV, Tsoukas GM, Thomsen AB, Kaltoft MS, Maislos M. Efficacy and safety of switching from sitagliptin to liraglutide in subjects with type 2 diabetes (LIRA-SWITCH): a randomized, double-blind, double-dummy, active-controlled 26-week trial. *Diabetes Obes Metab* 2016; **18**:1191-1198.

102. Rosenstock J, Aronson R, Grunberger G, Hanefeld M, Piatti P, Serusclat P, Cheng X, Zhou T, Niemoeller E, Souhami E, Davies M. Benefits of LixiLan, a Titratable Fixed-Ratio Combination of Insulin Glargine Plus Lixisenatide, Versus Insulin Glargine and Lixisenatide Monocomponents in Type 2 Diabetes Inadequately Controlled on Oral Agents: The LixiLan-O Randomized Trial. *Diabetes Care* 2016; **39**:2026-2035.

103. Rosenstock J, Guerci B, Hanefeld M, Gentile S, Aronson R, Tinahones FJ, Roy-Duval C, Souhami E, Wardecki M, Ye J, Perfetti R, Heller S. Prandial Options to Advance Basal Insulin Glargine Therapy: Testing Lixisenatide Plus Basal Insulin Versus Insulin Glulisine Either as Basal-Plus or Basal-Bolus in Type 2 Diabetes: The GetGoal Duo-2 Trial. *Diabetes Care* 2016; **39**:1318-1328.

104. Neal B, Perkovic V, Mahaffey KW, de Zeeuw D, Fulcher G, Erondu N, Shaw W, Law G, Desai M, Matthews DR. Canagliflozin and Cardiovascular and Renal Events in Type 2 Diabetes. *N Engl J Med* 2017; **377**:644-657.

105. Groop PH, Cooper ME, Perkovic V, Hocher B, Kanasaki K, Haneda M, Schernthaner G, Sharma K, Stanton RC, Toto R, Cescutti J, Gordat M, Meinicke T, Koitka-Weber A, Thiemann S, von Eynatten M. Linagliptin and its effects on hyperglycaemia and albuminuria in patients with type 2 diabetes and renal dysfunction: the randomized MARLINA-T2D trial. *Diabetes Obes Metab* 2017; **19**:1610-1619.

106. Holman RR, Bethel MA, Mentz RJ, Thompson VP, Lokhnygina Y, Buse JB, Chan JC, Choi J, Gustavson SM, Iqbal N, Maggioni AP, Marso SP, Ohman P, Pagidipati NJ, Poulter N, Ramachandran A, Zinman B, Hernandez AF. Effects of Once-Weekly Exenatide on Cardiovascular Outcomes in Type 2 Diabetes. *N Engl J Med* 2017; **377**:1228-1239.

107. Guja C, Frias JP, Somogyi A, Jabbour S, Wang H, Hardy E, Rosenstock J. Effect of exenatide QW or placebo, both added to titrated insulin glargine, in uncontrolled type 2 diabetes: The DURATION-7 randomized study. *Diabetes Obes Metab* 2018; **20**:1602-1614.
