# Supplementary material for: Comparative outcomes of heart failure among existent classes of anti-diabetic agents: a network meta-analysis of 171,253 participants from 91 randomized controlled trials
Source: Cardiovasc Diabetol. 2019 Apr 8;18:47. doi: 10.1186/s12933-019-0853-x (PMC6454617; doi:10.1186/s12933-019-0853-x)
Supplement: Supplementary file 5 — Additional file 5: Table S6. Potential inconsistency between direct and indirect comparisons assessed by the node-splitting method. [file 12933_2019_853_MOESM5_ESM.docx]

| Side | | Direct | | Indirect | | Difference | | |
| --- | --- | --- | --- | --- | --- | --- | --- | --- |
|  |  | Coef | Std.Err. | Coef. | Std.Err. | Coef. | Std.Err. | P>\|z\| |
| PLA | SGLT2i | -0.386 | 0.0735 | -0.485 | 0.697 | 0.0989 | 0.701 | 0.888 |
| PLA | SU | -0.575 | 1.188 | -0.00478 | 0.235 | -0.570 | 1.222 | 0.641 |
| PLA | TZD | -0.0466 | 0.639 | 0.478 | 0.283 | -0.524 | 0.703 | 0.456 |
| DPP4i | PLA | -0.0521 | 0.0592 | 0.0918 | 0.402 | -0.144 | 0.406 | 0.723 |
| DPP4i | GLP1a | 0.544 | 0.672 | -0.157 | 0.0891 | 0.702 | 0.678 | 0.301 |
| DPP4i | INS | 1.094 | 1.637 | -0.349 | 0.311 | 1.443 | 1.666 | 0.386 |
| DPP4i | MET | -1.678 | 1.587 | 0.105 | 0.302 | -1.783 | 1.608 | 0.268 |
| DPP4i | SGLT2i | -0.698 | 1.159 | -0.434 | 0.0930 | -0.264 | 1.163 | 0.820 |
| DPP4i | SU | -0.0167 | 0.334 | -0.135 | 0.312 | 0.119 | 0.457 | 0.795 |
| DPP4i | TZD | -0.189 | 0.943 | 0.386 | 0.268 | -0.574 | 0.985 | 0.560 |
| GLP1a | PLA | 0.0936 | 0.0685 | 0.208 | 0.463 | -0.114 | 0.469 | 0.807 |
| GLP1a | INS | 0.426 | 0.524 | -0.452 | 0.376 | 0.878 | 0.645 | 0.173 |
| GLP1a | MET | 0.933 | 1.229 | 0.148 | 0.310 | 0.784 | 1.267 | 0.536 |
| GLP1a | SU | -0.887 | 0.659 | 0.189 | 0.246 | -1.075 | 0.697 | 0.123 |
| GLP1a | TZD | 0.793 | 1.198 | 0.463 | 0.274 | 0.330 | 1.254 | 0.792 |
| INS | SU | 0.441 | 0.291 | -0.603 | 0.559 | 1.044 | 0.630 | 0.098 |
| MET | PLA | 1.395 | 2.005 | -0.133 | 0.301 | 1.528 | 2.028 | 0.451 |
| MET | SGLT2i | -0.358 | 1.640 | -0.491 | 0.310 | 0.133 | 1.669 | 0.937 |
| MET | SU | -0.351 | 0.308 | 0.131 | 0.336 | -0.481 | 0.465 | 0.300 |
| MET | TZD | 0.309 | 0.230 | 0.226 | 0.423 | 0.0836 | 0.480 | 0.862 |
| SGLT2i | SU | 0.386 | 0.767 | 0.353 | 0.249 | 0.0329 | 0.806 | 0.967 |
| SU | TZD | 0.455 | 0.171 | 0.224 | 0.401 | 0.232 | 0.434 | 0.593 |
