# Supplementary material for: Comparative outcomes of heart failure among existent classes of anti-diabetic agents: a network meta-analysis of 171,253 participants from 91 randomized controlled trials
Source: Cardiovasc Diabetol. 2019 Apr 8;18:47. doi: 10.1186/s12933-019-0853-x (PMC6454617; doi:10.1186/s12933-019-0853-x)
Supplement: Supplementary file 6 — Additional file 6: Figure S2. Sensitivity analysis after excluding studies with an arm of fewer than 100 patients. [file 12933_2019_853_MOESM6_ESM.docx]

| **SGLT2i** | 1.16 (0.62,2.15) | **1.34 (1.10,1.63)** | 1.44 (0.89,2.34) | **1.48 (1.28,1.71)** | 1.70 (0.91,3.17) | **1.54 (1.28,1.85)** | **2.21 (1.29,3.78)** |
| --- | --- | --- | --- | --- | --- | --- | --- |
| 0.86 (0.46,1.61) | **MET** | 1.16 (0.63,2.13) | 1.25 (0.75,2.07) | 1.28 (0.70,2.34) | 1.47 (0.75,2.85) | 1.34 (0.73,2.46) | **1.91 (1.06,3.43)** |
| **0.75 (0.61,0.91)** | 0.86 (0.47,1.58) | **INS** | 1.07 (0.67,1.73) | 1.10 (0.96,1.26) | 1.26 (0.68,2.34) | 1.15 (0.97,1.37) | 1.64 (0.97,2.79) |
| 0.69 (0.43,1.13) | 0.80 (0.48,1.33) | 0.93 (0.58,1.50) | **SU** | 1.03 (0.64,1.64) | 1.18 (0.76,1.83) | 1.07 (0.67,1.71) | **1.53 (1.12,2.09)** |
| **0.68 (0.59,0.78)** | 0.78 (0.43,1.44) | 0.91 (0.79,1.04) | 0.98 (0.61,1.56) | **DPP4i** | 1.15 (0.62,2.12) | 1.05 (0.93,1.17) | 1.49 (0.88,2.52) |
| 0.59 (0.32,1.10) | 0.68 (0.35,1.33) | 0.79 (0.43,1.47) | 0.85 (0.55,1.32) | 0.87 (0.47,1.61) | **PLA** | 0.91 (0.49,1.68) | 1.30 (0.87,1.94) |
| **0.65 (0.54,0.78)** | 0.75 (0.41,1.38) | 0.87 (0.73,1.03) | 0.93 (0.58,1.49) | 0.96 (0.85,1.08) | 1.10 (0.60,2.03) | **GLP1a** | 1.43 (0.85,2.41) |
| **0.45 (0.26,0.78)** | **0.52 (0.29,0.94)** | 0.61 (0.36,1.03) | **0.65 (0.48,0.89)** | 0.67 (0.40,1.13) | 0.77 (0.51,1.15) | 0.70 (0.41,1.18) | **TZD** |
