# Supplementary material for: Comparative outcomes of heart failure among existent classes of anti-diabetic agents: a network meta-analysis of 171,253 participants from 91 randomized controlled trials
Source: Cardiovasc Diabetol. 2019 Apr 8;18:47. doi: 10.1186/s12933-019-0853-x (PMC6454617; doi:10.1186/s12933-019-0853-x)
Supplement: Supplementary file 7 — Additional file 7: Figure S3. Sensitivity analysis after excluding studies with a follow-up period of fewer than 48 weeks. [file 12933_2019_853_MOESM7_ESM.docx]

| **SGLT2i** | 1.08 (0.54,2.14) | **1.34 (1.10,1.62)** | 1.43 (0.86,2.37) | **1.47 (1.27,1.69)** | 1.66 (0.88,3.14) | **1.56 (1.30,1.87)** | **2.15 (1.23,3.78)** |
| --- | --- | --- | --- | --- | --- | --- | --- |
| 0.93 (0.47,1.85) | **MET** | 1.24 (0.63,2.45) | 1.33 (0.78,2.26) | 1.36 (0.69,2.68) | 1.54 (0.78,3.07) | 1.45 (0.74,2.84) | **2.00 (1.08,3.70)** |
| **0.75 (0.62,0.91)** | 0.80 (0.41,1.59) | **INS** | 1.07 (0.65,1.76) | 1.10 (0.96,1.25) | 1.24 (0.66,2.34) | 1.16 (0.98,1.39) | 1.61 (0.92,2.81) |
| 0.70 (0.42,1.16) | 0.75 (0.44,1.28) | 0.94 (0.57,1.54) | **SU** | 1.03 (0.63,1.68) | 1.16 (0.75,1.81) | 1.09 (0.67,1.77) | **1.51 (1.10,2.07)** |
| **0.68 (0.59,0.79)** | 0.73 (0.37,1.44) | 0.91 (0.80,1.04) | 0.97 (0.60,1.59) | **DPP4i** | 1.13 (0.61,2.11) | 1.06 (0.94,1.19) | 1.47 (0.85,2.54) |
| 0.60 (0.32,1.14) | 0.65 (0.33,1.29) | 0.80 (0.43,1.51) | 0.86 (0.55,1.34) | 0.88 (0.47,1.65) | **PLA** | 0.94 (0.50,1.75) | 1.30 (0.87,1.93) |
| **0.64 (0.54,0.77)** | 0.69 (0.35,1.36) | 0.86 (0.72,1.02) | 0.92 (0.56,1.50) | 0.94 (0.84,1.06) | 1.07 (0.57,1.99) | **GLP1a** | 1.38 (0.80,2.40) |
| **0.46 (0.26,0.82)** | **0.50 (0.27,0.92)** | 0.62 (0.36,1.08) | **0.66 (0.48,0.91)** | 0.68 (0.39,1.18) | 0.77 (0.52,1.15) | 0.72 (0.42,1.25) | **TZD** |
